# Upcycling factory potato peel into a hierarchically porous carbon for dye removal, phytotoxicity mitigation, and wastewater remediation

**DOI:** 10.1039/d6ra02967a

**Published:** 2026-07-13

**Authors:** M. Bhavani Lakshmi, Alibasha Akbar, Paramita Pattanayak, Tanmay Chatterjee, Mihir Ghosh

**Affiliations:** a Department of Chemistry, SRM Institute of Science and Technology Kattankulathur 603203 Tamil Nadu India mihirg@srmist.edu.in; b Department of Chemistry, Birla Institute of Technology and Science, Pilani – Hyderabad Campus Jawahar Nagar, Kapra Mandal Hyderabad Telangana 500078 India

## Abstract

Potato peel from chip factories, a centralized, continuously generated agro-industrial waste stream, is valorized here into a nitrogen-enriched, hierarchically porous activated carbon (PMC) through melamine-assisted N-doping and KOH activation. Unlike laboratory-scale biomass studies relying on inconsistent feedstocks, this work leverages factory-scale peel sourcing to improve feedstock consistency and industrial relevance. The engineered carbon exhibits a micro–mesoporous architecture (BET surface area = 284.3 m^2^ g^−1^) facilitates molecular accessibility, together with a highly super-hydrophilic surface (contact angle = 3.7°) and a strongly negative surface charge (*ζ* = −35.6 mV; pH_pzc_ = 6.8). XPS, FTIR, Raman, and surface analyses show abundant nitrogen and oxygen containing functionalities and a defect-rich carbon matrix that facilitates adsorption through pore filling, hydrogen bonding, π–π interactions, and electrostatic attractions. PMC rapidly removes 98.9% of crystal violet in 60 min (Langmuir *q*_m_ = 110.18 mg g^−1^, *R*^2^ = 0.99) and follows pseudo-second-order kinetics (*R*^2^ = 0.99). The adsorbent exhibits clear selectivity for cationic over anionic dyes, achieving 96.9% and 87% decolourization of two real textile effluents within 1 h, and retains >89% of its capacity after five ethanol regeneration cycles, demonstrating structural stability and practical reusability. Treated effluent restores germination and seedling growth in *Vigna radiata*, demonstrating an adsorption-based ecological remediation pathway. These results highlight the potential of PMC as an efficient and reusable adsorbent for practical dye removal from industrial wastewater. By coupling factory-scale feedstock consistency with targeted heteroatom tuning and pore design, PMC offers a low-cost, potentially scalable route for selective dye remediation aligned with circular-economy goals.

## Abstract 2

Potato peel from chip factories, a centralized, continuously generated agro-industrial waste stream, is valorized here into a nitrogen-enriched, hierarchically porous activated carbon (PMC) through melamine-assisted N-doping and KOH activation.

## Graphical abstract

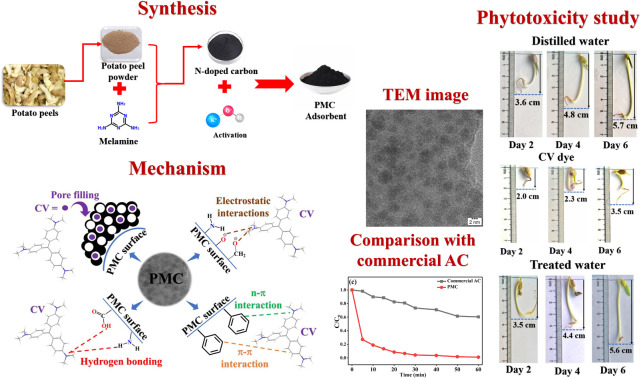

## 1. Introduction

Potato (*Solanum tuberosum*) holds a strategic position in India's agro-industrial landscape, combining importance for food security with the rapid expansion of processing sectors for chips, dehydrated foods, and ready-to-eat foods. As the world's second-largest producers, this industrial growth results in the centralized, continuous generation of potato peel residues.^1–3^ Activated carbon derived from biomass continues to dominate adsorption-based wastewater treatment owing to their tunable porosity and surface chemistry. However, many lignocellulosic feedstocks commonly studied in the literature (rice husk,^4^ coconut shell,^5^ sawdust,^6^ nut shells^7^) are constrained by high lignin content, mineral ash (especially silica), and inherent hydrophobicity, which collectively hinder homogeneous chemical impregnation, slow aqueous wetting, and limit accessible pore development.^8^ Crystal Violet (CV) is a toxic triphenylmethane cationic dye commonly utilized in textile and industrial applications. CV-contaminated wastewater is a major environmental problem because it may penetrate biological cells and cause adverse health effects, including skin and eye irritation.^9–12^ Several methods for treating water, including membrane filtration, coagulation, ion exchange, reverse osmosis, and photo-Fenton oxidation, have been studied;^13–19^ however, many face high costs and practical challenges. Because of its effectiveness, ease of use, and capacity to employ inexpensive adsorbents derived from waste, adsorption remains one of the most successful methods.^13^ In contrast, potato peel (PP) exhibits a biochemical architecture fundamentally distinct from lignified residues. It is dominated by starch- and pectin-rich polysaccharides and contains comparatively low lignin and silica fractions.^14^ This composition confers intrinsic hydrophilicity and high wettability, enabling efficient penetration of activating agents and nitrogen precursors during chemical processing. During carbonization and chemical activation, the oxygen-rich polymer matrix of PP facilitates the formation of abundant surface functional groups while promoting controlled pore development without excessive structural collapse. As a result, PP-derived carbons are predisposed to form aqueously accessible micro–mesoporous networks, a feature that is particularly advantageous for the adsorption of dissolved organic contaminants. Our work bridges that gap by integrating factory-level feedstock with a deliberate heteroatom pore engineering protocol. Building on these advantages, we introduce a factory-to-filter valorization strategy in which potato-chip peel is converted into a nitrogen-doped, hierarchically porous activated carbon (PMC) designed specifically for the selective removal of cationic dyes. The synthesis integrates melamine-assisted nitrogen doping with KOH-mediated activation, leveraging complementary mechanisms for heteroatom incorporation and pore engineering. Melamine acts as both a nitrogen source and a gas-evolving porogen, generating pyridinic and pyrrolic nitrogen species and defect sites that enhance electrostatic attraction and Lewis base interactions.^15–17^ Concurrently, KOH activation yields interconnected micro–mesoporous architectures that maximize surface accessibility and reduce diffusion barriers.^18,19^

Crucially, none have addressed the industrial reality of chip-factory peel, in which batch-to-batch consistency and year-round availability enable scalable production. This work introduces three key innovations by specifically targeting potato chip factory peel as a feedstock and employing a melamine–KOH tuning strategy. First, it demonstrates the value of an underutilized, factory-scale biomass as an industrially relevant precursor material. Second, it establishes a tailored synthesis that couples nitrogen chemistry with hierarchical porosity to favor cationic dye selectivity. Third, it validates the material's practical utility *via* real-effluent tests, regenerability, and biological (phytotoxicity) mitigation. Together, these elements position PMC as a scalable, low-cost, and environmentally compatible adsorbent candidate for selective dye remediation and for integration into circular-economy wastewater treatment schemes, warranting further techno-economic evaluation for industrial adoption.

## 2. Experimental section

### 2.1. Materials

PP was collected from the potato chips manufacturing factory and used as the key material for producing activated carbon. Melamine (SRL Chemicals, 99%, India), potassium hydroxide (KOH, SRL Chemicals, ≥85%, India), potassium dihydrogen orthophosphate (SRL Chemicals, 99.5% extrapure, India), anhydrous potassium phosphate dibasic (SRL Chemicals, ≥99.5%, India), hydrochloric acid (HCl, SRL Chemicals, India), sodium hydroxide (SRL Chemicals, India), and ethanol (SRL Chemicals, ≥85%, India) were used directly without further purification. Crystal violet (colour index: 42555, SRL Chemicals, India) was used as the model cationic dye for adsorption studies. A stock dye solution (1000 mg L^−1^) was prepared by dissolving 1000 mg of CV in 1 L of deionized water and diluted as needed; pH was adjusted using 0.1 M NaOH or HCl.

### 2.2. Compositional analysis of PP

The compositional characteristics of PP were evaluated to confirm its consistency and suitability as a biomass precursor. Experimental proximate analysis of the PP feedstock showed moisture, ash, volatile matter, and fixed carbon contents of 8.5%, 9.79%, 72.4%, and 9.3%, respectively. In addition, previously reported elemental and biochemical composition data from the literature indicate the presence of carbon-rich and oxygenated constituents, including starch, lignocellulosic polysaccharides, and lignin fractions.^20^ The high volatile matter content and oxygen-rich composition suggest favorable thermochemical reactivity and pore-forming ability during pyrolysis and KOH activation. Detailed compositional, elemental, and calorific value analyses of PP are provided in the SI (Table S1).

### 2.3. Preparation of nitrogen-doped activated carbon derived from PP biomass *via* KOH activation

**Fig. 1:**
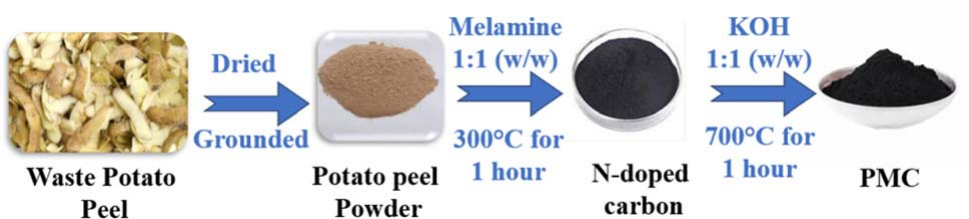
Preparation of activated carbon derived from PP using two-step activation.

Potato peels (PP) collected were thoroughly rinsed with deionized water, dried in an oven at 100 °C for 24 hours, and then ground to a fine powder. Using an agate mortar, 1 g of PP powder and 1 g of melamine (1 : 1, w/w) were physically combined for nitrogen doping without the use of a solvent. In a muffle furnace, the homogeneous mixture was pre-carbonized for 1 h at 300 °C (heating rate: 10 °C min^−1^).^21,22^ The resulting nitrogen-enriched carbon precursor was gathered once it had cooled. One gram of solid KOH (1 : 1, w/w) and one gram of the N-doped precursor were physically mixed before being placed in a tubular furnace for activation. At a heating rate of 10 °C min^−1^, thermal activation was performed for one hour at 700 °C. The product cooled to room temperature without external cooling. Before being rinsed five times with deionized water until the pH was neutral (∼7), the active material was cleaned with 0.01 M HCl. The solid was then finely ground after being oven-dried for 12 hours at 100 °C. PMC stands for the final nitrogen-doped, KOH-activated porous carbon. The overall synthesis procedure is illustrated in Fig. 1.

### 2.4. Characterizations

Powder X-ray diffraction (XRD) patterns were recorded on a Panalytical X'Pert Pro diffractometer (2*θ* = 10°–80°, Cu Kα, *λ* = 0.154 nm). Fourier-transform infrared (FTIR) spectra were collected in the 4000–400 cm^−1^ range using a Bruker Alpha T spectrometer (ATR mode). Morphology and elemental composition were examined by FESEM-EDS (FEI Apreo S) and HRTEM (JEOL 2010F, 200 kV), while surface topography was analyzed using AFM (JPK-NANOWIZARD 4). Nitrogen adsorption–desorption isotherms were measured on a Microtrac BELSORP mini II to determine BET surface area, pore size, and volume. XPS (PHI VersaProbe III, Al Kα) and Raman spectroscopy (HORIBA LabRAM HR Evolution, 532 nm) were used for surface chemistry and structural analysis. Particle size and zeta potential were measured by DLS (Malvern ZS-3600). Adsorption studies were performed using a UV-vis spectrophotometer (Agilent Cary 60), and the point of zero charge (pH_pzc_) and surface functional groups were determined *via* the solid-addition and Boehm titration methods, respectively.

## 3. Results and discussion

### 3.1. Characterizations of adsorbents derived from *Solanum tuberosum*

**Fig. 2:**
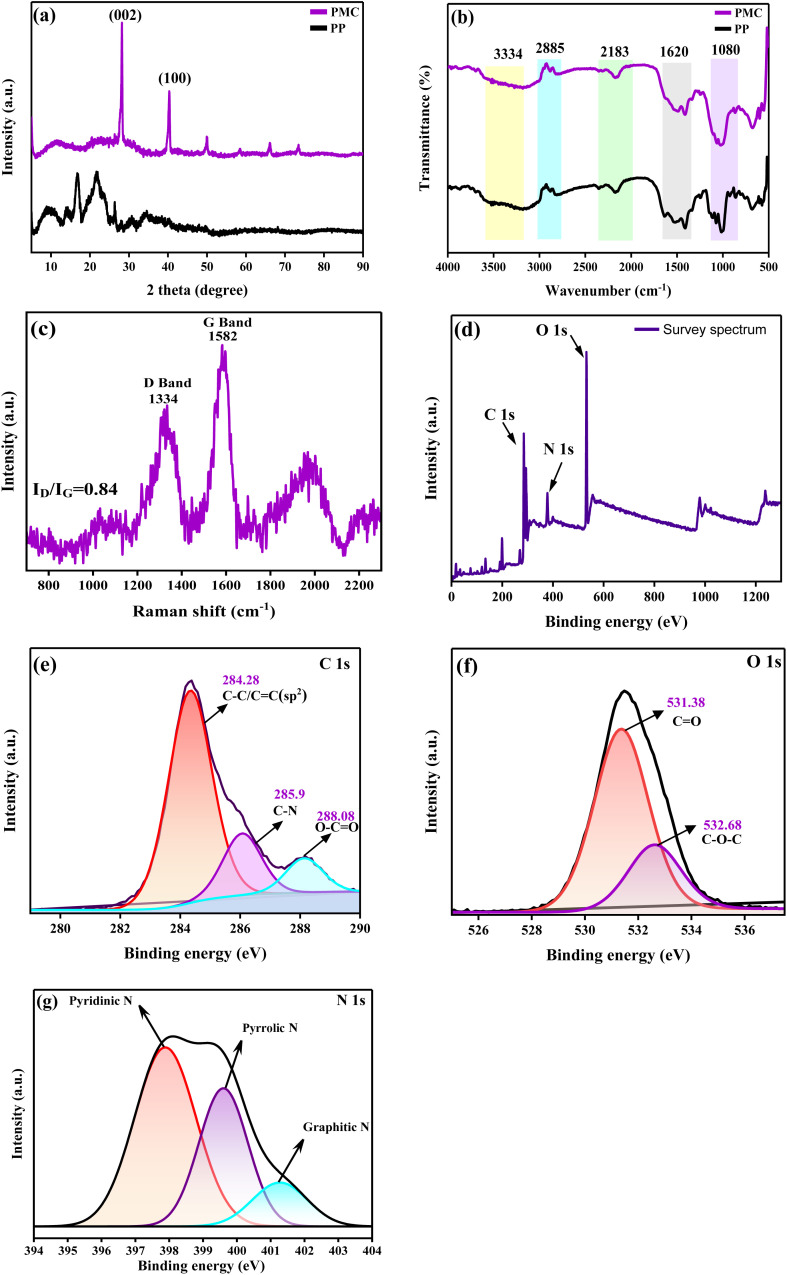
(a) XRD pattern of PP and PMC, (b) FTIR spectrum of PP and PMC, (c) Raman spectrum of PMC, XPS spectra of PMC activated carbon: (d) survey spectra, (e) C 1s core level spectra, (f) O 1s core level spectra, and (g) N 1s core level spectra.

To understand the relationship between structural properties and adsorption performance, the synthesized PMC adsorbent was comprehensively characterized using multiple analytical techniques. Structural, morphological, and surface chemical properties were evaluated to identify the active adsorption sites and pore architecture responsible for dye removal efficiency. Fig. 2a shows the XRD patterns of PP and the synthesized PMC, which reveal the phase composition and structural properties of the PMC. The (002) and (100) planes of partly graphitic carbon are responsible for the two large diffraction peaks observed at approximately 28° and 40°, respectively.^23^ The broad peaks and lack of crystalline features indicate that the carbon is primarily amorphous, with little graphitization. Notably, the graphitic domains are disrupted by structural flaws introduced by KOH activation and nitrogen doping from melamine. The material has a disordered, defect-rich structure that is ideal for adsorption applications. At the same time, the peak intensity and sharpness indicate that the carbon framework retains its original order.^24^

The Fourier transform infrared (FTIR) spectra for the PP and PMC samples are illustrated in Fig. 2b. Both materials display unique vibrational characteristics associated with specific surface functional groups. The absorption band centered around 3334 cm^−1^ is attributed to the O–H stretching vibration of hydroxyl groups and physically adsorbed water molecules. The absorption peaks at 2920 cm^−1^ and 2885 cm^−1^ are attributed to the asymmetric and symmetric stretching vibrations of aliphatic C–H bonds, respectively. The band observed around 1620 cm^−1^ is associated with C=C stretching in aromatic domains and/or C=O stretching in carbonyl functionalities, suggesting the presence of conjugated carbon structures. The absorption near 1080 cm^−1^ is attributed to C–O stretching vibrations related to phenolic or alcoholic groups. A comparison of the spectra for PP and PMC 700 indicates a significant reduction in the intensity of oxygen-containing functional groups following activation, highlighting the successful removal of surface oxygen species during high-temperature treatment. The appearance of more distinct absorption features in the PMC sample indicates improved graphitization and structural ordering, thereby affirming the effective chemical activation and carbonization of the precursor material at 700 °C.

Fig. 2c shows the corresponding Raman spectrum used to assess the degree of graphitization and structural ordering of the synthesized PMC carbon material. Two distinct bands are identified at approximately 1334 cm^−1^ and 1582 cm^−1^, corresponding to the D and G bands, respectively. The Raman spectrum of PMC has two distinctive bands centered at 1334 cm^−1^ (D band) and 1582 cm^−1^ (G band). *I*_D_/*I*_G_, the calculated intensity ratio, is 0.84. A relatively high degree of graphitic ordering with a low defect density is indicated by an *I*_D_/*I*_G_ value less than unity. The partially graphitized carbon domains and defect sites produced during nitrogen incorporation and KOH activation have been identified by the ratio of 0.84. This structure enhances surface reactivity and electronic conductivity by providing an optimal balance between ordered sp^2^ carbon networks and defect-rich regions. Additionally, the low number of defects suggests that activation facilitated the formation of graphitic pores without causing severe structural disturbance. The results correspond with the FTIR and XRD analyses, confirming the presence of both amorphous and graphitic regions within the activated carbon framework.

XPS measurements were conducted to examine the surface elemental composition of the PMC. Fig. 2d–g presents the XPS spectra of the activated carbon. The survey spectrum (Fig. 2d) confirms the presence of C 1s, O 1s, and N 1s signals, verifying the successful incorporation of nitrogen into the carbon matrix. The C 1s spectrum (Fig. 2e) can be deconvoluted into three peaks at 284.28 eV (C–C/C=C), 285.9 eV (C–N), and 288.08 eV (O–C=O), indicating the coexistence of graphitic carbon, nitrogen, and oxygen species. The O 1s spectrum (Fig. 2f) exhibits peaks at 531.38 eV (C=O) and 532.68 eV (C–O–C), revealing oxygen functionalities that enhance surface polarity and adsorption affinity. There is strong concordance between the XPS analysis and the FTIR readings. The functional groups identified by FTIR, namely O–H (∼3334 cm^−1^), C–H (2920 and 2885 cm^−1^), C=C/C=O (∼1620 cm^−1^), and C–O (∼1080 cm^−1^), correspond with the oxygen- and carbon-related surface species indicated by the XPS O 1s and C 1s spectra (C–N, C=O, and C–O–C). The decreased oxygen-containing band intensity in the PMC sample's FTIR spectrum is consistent with XPS data showing decreased surface oxygen functionalities following high-temperature activation, and the increased graphitic character observed in FTIR supports the C–C/C=C contribution that predominates in the XPS C 1s spectrum. Taken together, these additional findings support successful chemical activation, heteroatom incorporation, and surface functionalization of the activated carbon. For the high-resolution N 1s spectrum (Fig. 2g), three different nitrogen structures were identified by deconvolution. Pyridinic-N is responsible for the peak at about 397.8 eV, which increases electron-donating capacity and adds one p-electron to the aromatic π-system. Pyrrolic-N is the component at approximately 399.6 eV and is typically associated with five-membered heterocycles. Graphitic-N (quaternary N) is responsible for a strong signal centered at approximately 401.4 eV, which indicates nitrogen substitution within the graphitic carbon framework. The presence of graphitic and pyridinic nitrogen among these species indicates that stable nitrogen functionalities were successfully introduced into the carbon matrix by melamine breakdown during high-temperature KOH activation. It is well known that graphitic-N and pyridinic-N enhance electron density distribution and generate active sites, both of which are advantageous for adsorption processes.^25^

Surface wettability is an important factor influencing the interactions between adsorbents and aqueous contaminants. Surfaces are classified as super-hydrophilic (*θ* < 10°), hydrophilic (10° < *θ* < 90°), hydrophobic (90° < *θ* < 150°), or super-hydrophobic (*θ* > 150°) based on contact angle (*θ*) measurements; lower angles indicate higher polarity and water affinity. Drop contour analysis confirmed the hydrophilic nature of virgin PP by revealing a contact angle of 11.8° (ref. 26 and 27) (Fig. S1a). The contact angle further decreased to 3.7° for PMC after melamine and KOH activation (Fig. S1b), indicating a transition to a super-hydrophilic surface. The introduction of numerous oxygen-containing functional groups and the enhanced surface roughness induced by alkali activation are responsible for this notable improvement in wettability. Due to the enhanced accessibility of active sites and their super-hydrophilic nature, PMC exhibits excellent dye adsorption performance under solution conditions.

**Fig. 3:**
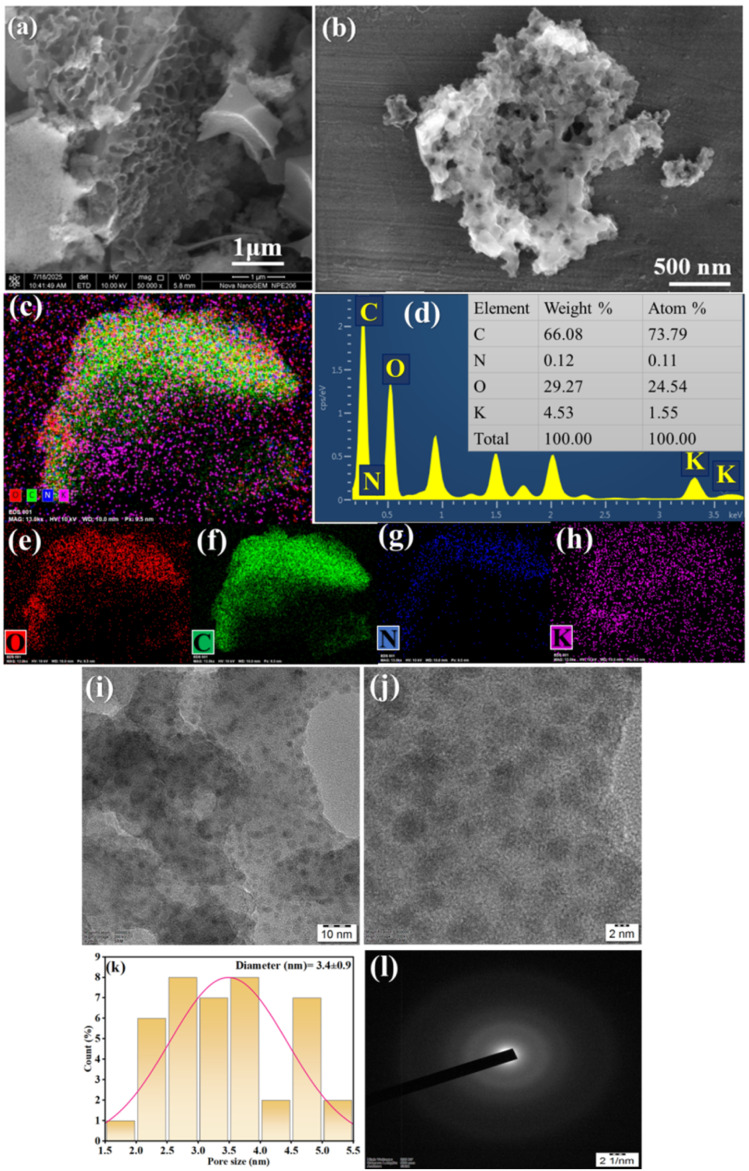
(a and b) SEM images of PMC samples, (c) overall elemental mapping of PMC samples, (d) EDS spectrum, (e) elemental mapping of O (red colour), (f) elemental mapping of C (green colour), (g) elemental mapping of N (blue colour), (h) elemental mapping of K (violet colour), (i and j) TEM images of PMC samples, (k) pore size analysis, (l) SAED pattern of PMC sample.

Fig. 3a and b shows that the PMC derived from PP develops a rough, irregular surface with abundant voids and interconnected channels, evidencing a well-developed hierarchical porous architecture. Higher-magnification images confirm a dense distribution of micro- and mesopores throughout the carbon framework. This morphology favors rapid intraparticle diffusion and effective exposure of adsorption sites to dye molecules, as shown in the overall elemental mapping in Fig. 3c. The EDS spectrum (Fig. 3d and S2a) indicates that the material is primarily composed of carbon, oxygen, and nitrogen, with a small amount of potassium, consistent with a biomass origin and KOH-based activation. The high carbon content reflects the formation of a stable carbonaceous matrix, whereas the significant oxygen and nitrogen fractions imply heteroatom-doped surface functionalities that enhance surface polarity, generate additional active sites, and strengthen interactions with cationic dyes; the weak potassium signal suggests residual K species that may further contribute to surface basicity and pore development, and the elemental mapping was shown in Fig. 3e–h. AFM was used to further investigate surface topography, in addition to the SEM results (Fig. S2b). The AFM 3D image shows a rough, heterogeneous surface with uniformly distributed protrusions and valleys across the scanned area (10 × 10 µm^2^). The SEM results are supported by these nanoscale surface imperfections, which validate the development of a porous and textured morphology.

TEM images (Fig. 3i and j) further corroborate the presence of a micro/mesoporous network, with an average pore diameter of about 3.8 nm, as illustrated in Fig. 3k, confirming the predominantly mesoporous nature of the PMC. The corresponding SAED pattern (Fig. 3l) exhibits broad diffuse rings, revealing an amorphous carbon structure rich in defect sites, which is typically beneficial for adsorption processes. Overall, the combination of hierarchical mesoporosity, an amorphous structure, and heteroatom-containing surface groups is expected to facilitate rapid pore diffusion and a high density of active sites, thereby enhancing adsorption capacity and kinetics for cationic dye removal from wastewater.

**Fig. 4:**
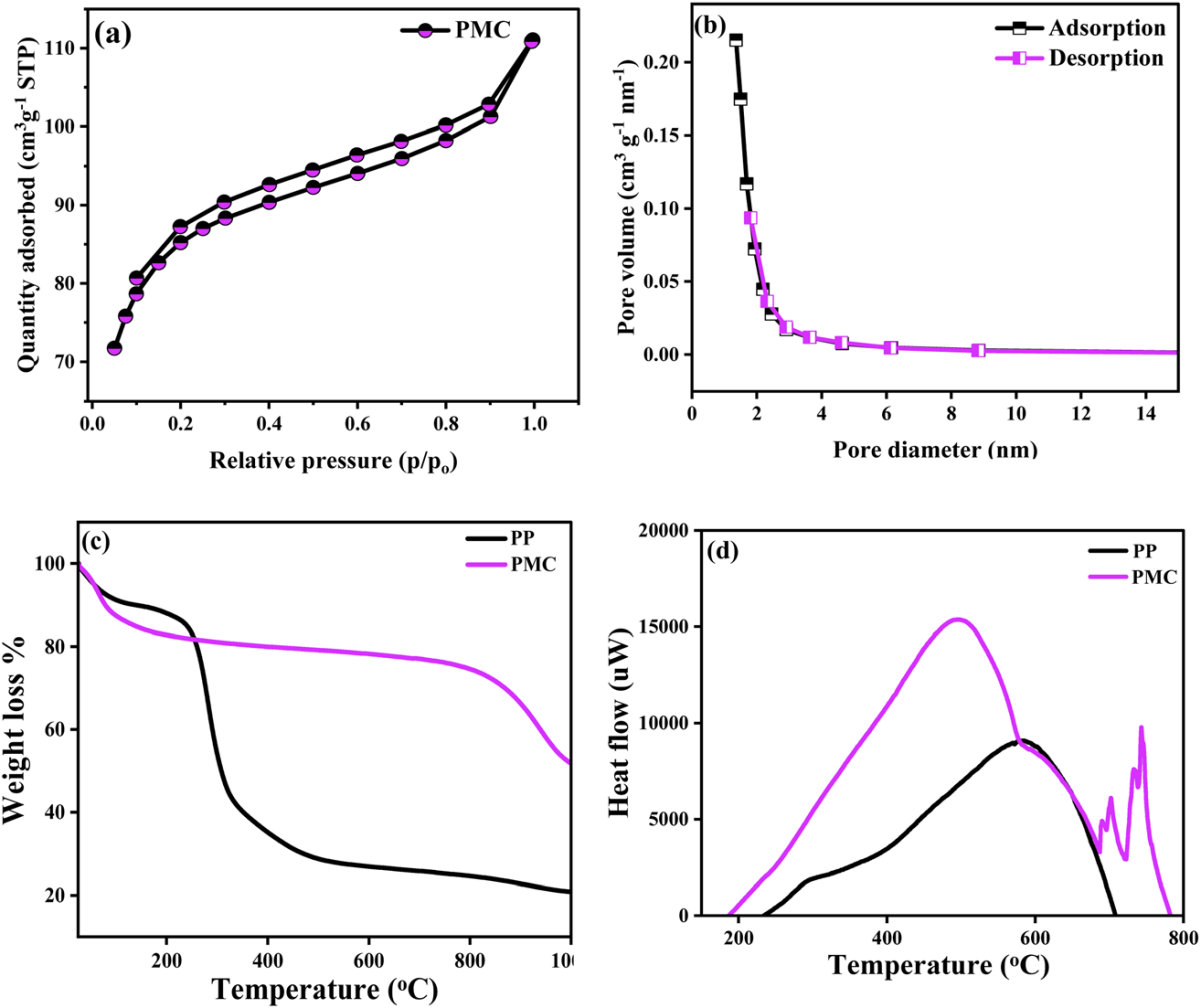
(a) BET isotherm of PMC nanocomposite and (b) BJH plot, (c) TGA spectrum of PP and PMC, and (d) DSC spectrum of PP and PMC.

The textural properties of the melamine-doped KOH-activated carbon (PMC) were evaluated using N_2_ adsorption–desorption measurements at 77 K, as shown in Fig. 4a and b. The creation of a mostly mesoporous structure with the existence of tiny slit-like pores is confirmed by the adsorption–desorption isotherm's type IV(a) profile with a prominent H4 hysteresis loop. These isotherm features are common to carbon compounds made from biomass that have been chemically activated and doped with heteroatoms. In mesoporous adsorbents, such as oxide gels, industrial adsorbents, and mesoporous molecular sieves, where adsorption occurs *via* monolayer–multilayer synthesis and capillary condensation within mesopores, type IV isotherms are prominent. The observed H4 hysteresis loop suggests the coexistence of micropores and narrow mesopores. These pores are typically observed in carbonaceous materials with slit-shaped morphology arising from stacked carbon layers. In the present scheme, melamine doping and KOH activation act in concert to control pore development. Melamine serves as a nitrogen source and contributes to structural flaws and pore stabilization, whereas KOH promotes extensive pore etching and widening.^28^ The Brunauer–Emmett–Teller (BET) analysis indicates that PMC has a specific surface area of 284.3 m^2^ g^−1^ and a total pore volume of 0.1718 cm^3^ g^−1^, suggesting the effective development of a porous framework. While the large pore volume and mesoporous architecture enable effective mass transfer and diffusion of adsorbate molecules, the comparatively high surface area provides numerous accessible adsorption sites.

Fig. 4c presents thermogravimetric analysis (TGA) and differential scanning calorimetry (DSC) data for PP and PMC, which elucidate their thermal behavior and justify the selected carbonization temperature. The PP sample exhibits an initial weight loss below approximately 150 °C, attributable to the elimination of physically adsorbed moisture and volatile compounds. A major mass loss occurring between about 200 and 400 °C is associated with the decomposition of highly labile organic compounds. This is followed by a gradual degradation of more stable carbonaceous materials at elevated temperatures, resulting in a comparatively low char yield. In contrast, PMC exhibits substantially improved thermal stability, characterized by minimal weight loss at lower temperatures and a greater decomposition range shifted to higher temperatures. KOH activation results in a more condensed and thermally stable carbon framework, as evidenced by the increased residual mass retained up to 1000 °C. The TGA results are further supported by the DSC profiles shown in Fig. 4d. Both samples exhibit overall exothermic behavior, attributable to the combustion of the organic component and thermal breakdown. Importantly, PMC exhibits a longer and stronger exothermic reaction at higher temperatures, with pronounced thermal transitions around 600–700 °C, indicating stability, aromatization, and carbon-structure rearrangement during activation. These findings led to the conclusion that 700 °C is the optimal carbonization temperature, as it ensures the complete breakdown of unstable organic moieties while increasing structural order and improving char yield without excessive carbon burn-off. This temperature offers an optimal equilibrium among thermal stability, carbon framework formation, and material integrity for future adsorption applications.

The zeta potential was measured at neutral pH (pH = 7) (Fig. S3a) and yielded −35.6 mV, indicating a strongly negative surface charge that provides significant electrostatic stabilization to the suspension. The significant negative potential is attributed to surface oxygen-containing functional groups, which inhibit particle aggregation through electrostatic repulsion and facilitate favorable interactions with cationic dye molecules through electrostatic attraction and ion–dipole interactions. The characteristics collectively highlight the potential of the synthesized material for efficient adsorption-based water purification.

Dynamic light scattering (DLS) analysis (Fig. S3b) indicated that the synthesized sample had a *Z*-average hydrodynamic diameter of 563.5 nm and a polydispersity index (PDI) of 0.352, suggesting a moderately uniform particle size distribution. The prominent peak at 494.8 nm indicates that most particles are effectively dispersed at the nanoscale, whereas a small fraction (3.4%) corresponds to larger aggregates near 540 nm. A moderate PDI value indicates sufficient colloidal stability and dispersion uniformity. Smaller and more uniformly distributed particles typically increase surface area and interfacial activity, thereby favoring adsorption processes and surface-mediated interactions.

**Fig. 5:**
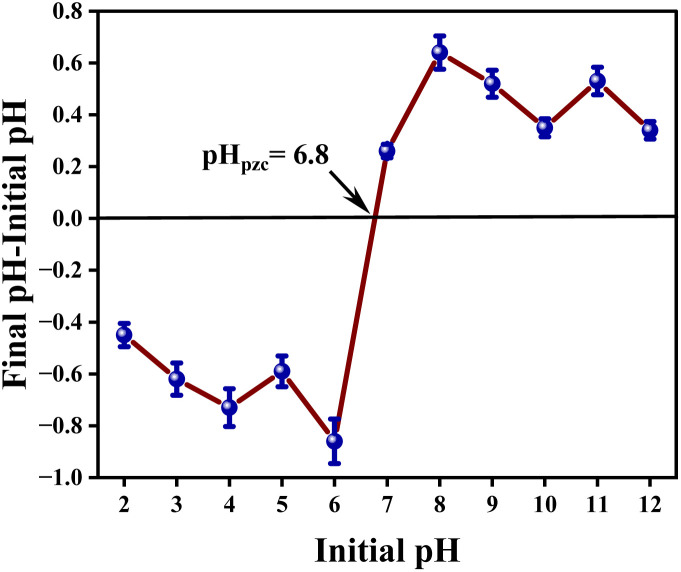
Point of zero charge of PMC.

The difference in pH (ΔpH) between the initial (pH_i_) and final (pH_f_) values was plotted against pH_i_ to identify the point of zero charge (pH_pzc_), indicated by the intersection of the curve with the axis, as shown in Fig. 5. The PMC composite exhibited a pH_pzc_ of 6.8, indicating that its surface becomes negatively charged at pH values above this value, thereby facilitating the adsorption of cationic dyes such as CV. At pH values below the pH_pzc_, the surface is positively charged, whereas at pH values above the pH_pzc_, the surface is negatively charged. The pH_pzc_ denotes the pH level at which the zeta potential equals zero, indicating a neutral net surface charge. The behavior of surface charge significantly affects electrostatic interactions and determines the adsorbent's affinity for ionic pollutants.

The Boehm titration method was used to fully evaluate the surface chemistry of activated carbon derived from PP and to determine the distribution of basic and acidic functional groups. However, before calculating surface basicity and acidity, several assumptions were made. HCl can neutralize all basic groups, whereas only acidic groups are neutralized by NaOH, Na_2_CO_3_, and NaHCO_3_. Table 1 indicates that the activated carbon has significant levels of lactonic (0.0201 mmol g^−1^), phenolic (0.0362 mmol g^−1^), and carboxyl (0.0479 mmol g^−1^) groups, totaling 0.1042 mmol g^−1^. However, the basicity was 0.08375 mmol g^−1^, indicating that the surface is predominantly acidic despite the presence of both basic and acidic functionalities. The incorporation of oxygenated functional groups during activation is often associated with the acidic nature of biomass-derived activated carbons. The abundance of oxygenated acidic functionalities enhances the adsorbent's ability to interact with cationic pollutants through electrostatic attraction, hydrogen bonding, and other surface-mediated mechanisms. More acidic groups indicate a greater number of oxygen-containing functional groups, which is why cationic dyes exhibit higher adsorption.^29^ Therefore, PMC is going to perform as an effective adsorbent for cationic dyes.

**Table 1:** Determination of acidic and basic groups using the Boehm titration method

Boehm's titration method					
PMC	Carboxyl groups	Lactone	Phenolic	Acidity	Basicity
Adsorbed (mmol g^−1^)	0.0479	0.0201	0.0362	0.1042	0.08375

### 3.2. Adsorption behaviour of the synthesized adsorbent

**Fig. 6:**
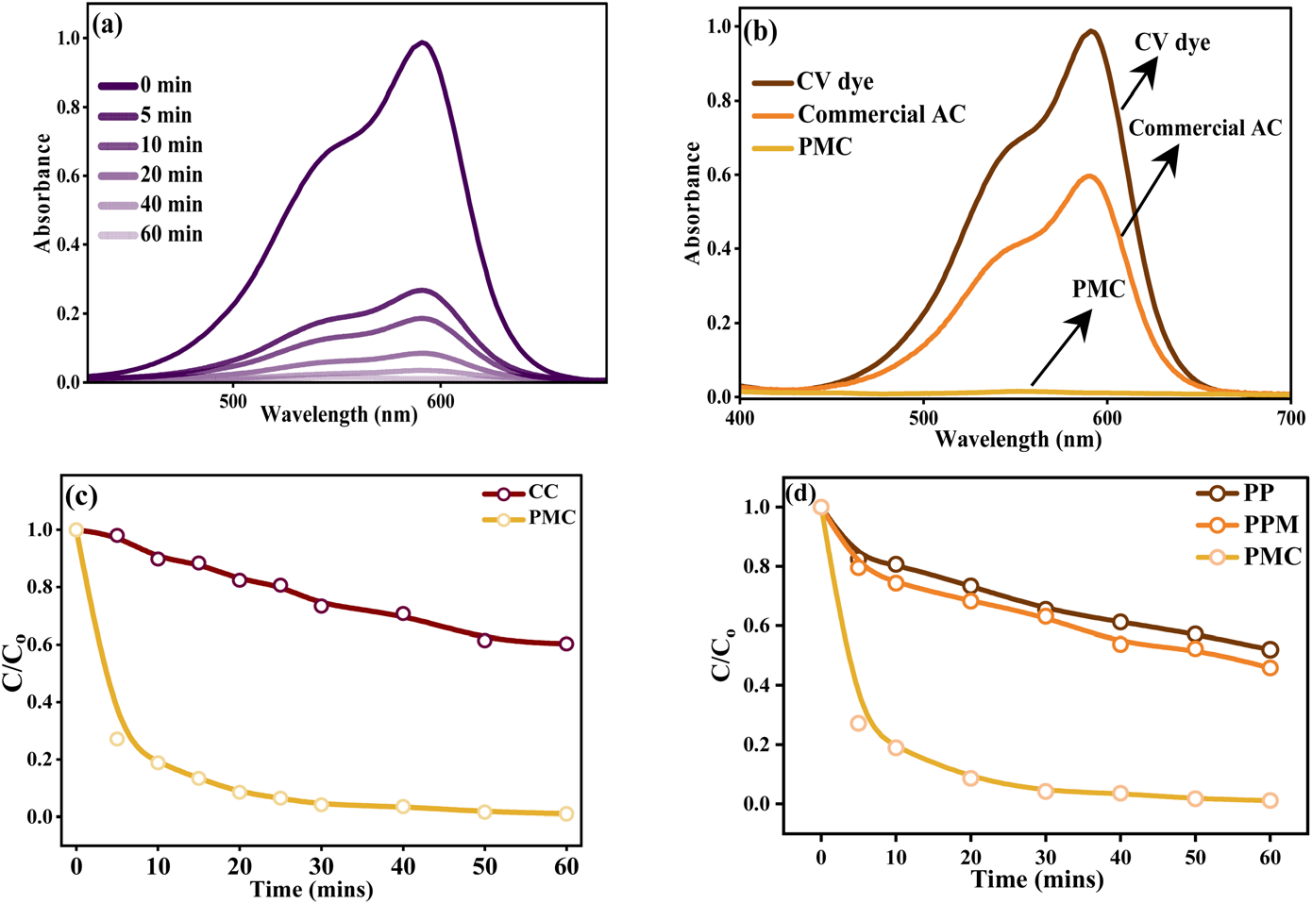
(a) UV-vis spectra depicting the adsorption of CV dye in the presence of PMC, (b) UV-visible spectra of CV after treatment with CC and PMC in 60 minutes, and (c) time-dependent adsorption of CV on CC and the PMC adsorbent, (d) time-dependent *C*/*C*_0_ profiles for CV adsorption over PP, PPM, and PMC illustrating comparative uptake efficiencies over time.

The maximum absorption peak of CV was recorded at *λ*_max_ = 590 nm (Fig. 6a). The addition of the PMC composite resulted in a significant decrease in the absorbance intensity of CV with a prolonged contact time, which became nearly negligible within 60 minutes, suggesting a rapid and effective removal of the dye. The lack of the characteristic absorption peak indicates near-complete decolorization (98.9%) of the violet dye solution, thereby confirming the strong affinity and high adsorption capacity of the PMC adsorbent for CV. These findings further demonstrate the PMC composite's high efficiency and selectivity in removing cationic dyes from aqueous solutions. Commercial activated carbon (CC) and the synthesized PMC adsorbent were used to assess the adsorption performance of CV (Fig. 6b). The untreated CV solution exhibited a characteristic absorption band that showed only a minimal decrease after treatment with commercial activated carbon, indicating limited dye adsorption capacity. The CV absorption peak, by contrast, almost completely vanished after PMC treatment, with absorbance approaching the baseline. The observed spectral reduction indicates that PMC exhibits significantly higher adsorption efficiency than commercial activated carbon, highlighting its strong affinity for cationic dyes. In Fig. 6c, the time-dependent adsorption behavior of CV on CC and PMC is shown. PMC exhibited a sudden and sustained decrease in the *C*/*C*_0_ ratio over the adsorption period, indicating rapid uptake kinetics and enhanced surface interactions with CV molecules. In comparison, CC exhibited a slower and smaller reduction in *C*/*C*_0_, indicating reduced adsorption performance. PMC consistently maintained lower *C*/*C*_0_ values than CC across all phases of the contact time, indicating a higher adsorption capacity. These findings show that effective CV removal from aqueous solutions is made practicable by PMC's enhanced surface functionality and porous structure.

The adsorption behavior of CV on PP, melamine-modified potato peel (PPM), and PMC is shown in Fig. 6d. There is a noticeable improvement in adsorption performance with each modification stage. Due to its small surface area and lack of active binding sites, pristine PP exhibited the smallest reduction in the *C*/*C*_0_ ratio across the entire contact time, indicating limited adsorption capacity. A greater decrease in *C*/*C*_0_ was observed after melamine (PPM) treatment, indicating the addition of nitrogen-containing functional groups that strengthen dye–adsorbent interactions and enhance adsorption efficiency. PMC demonstrated the best performance, with KOH activation accelerating the *C*/*C*_0_ drop, particularly in the first few minutes of contact. Improved surface area, porosity, and a higher density of accessible active sites are evidenced by rapid uptake and a significantly reduced residual concentration. The enhanced adsorption kinetics and capacity for CV removal result from the synergistic effects of sequential melamine functionalization and KOH activation on surface chemistry and structure. Overall, the adsorption efficiency follows the order PP < PPM < PMC.

### 3.3. Adsorption parameters

#### 3.3.1. Effect of pH

**Fig. 7:**
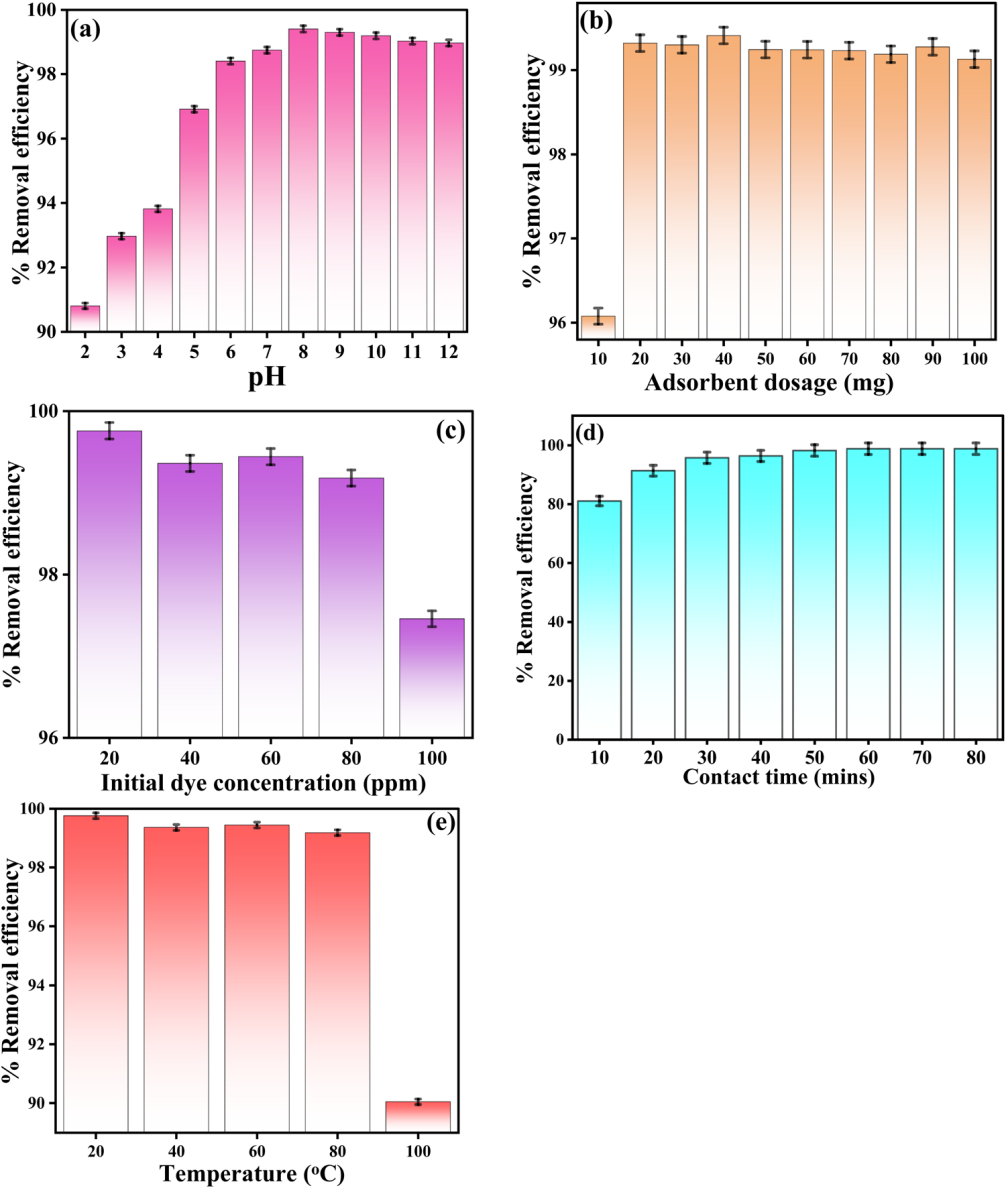
(a) Effect of pH, (b) effect of adsorbent dosage, (c) effect of initial dye concentration, (d) effect of contact time, and (e) effect of temperature.

The effect of pH on CV adsorption by PMC was investigated between 2 and 12 (Fig. 7a). The surface charge of PMC is controlled by its pH_pzc_, which is 6.8, as shown in Fig. 5. When the pH is below 6.8, the surface of PMC becomes positive because of protonation of surface groups. When the pH is above 6.8, the surface becomes negative because of deprotonation, confirmed by the zeta potential of −35.6 mV at pH 7 (Fig. S3a). At pH 2–4, removal efficiency ranged from 90.8% to 96.91%. Despite the positive surface charge electrostatically repelling cationic CV, high removal is maintained by pH-independent non-electrostatic mechanisms: π–π stacking between PMC's graphitic domains (Raman *I*_D_/*I*_G_ = 0.84; XPS C 1s: 284.28 eV) and CV's phenyl rings, and hydrogen bonding between CV's dimethylamino groups and surface –OH and C=O functionalities (FTIR: 3334 and 1620 cm^−1^). At pH 6, removal exceeds 98% despite near-zero surface charge, confirming that non-electrostatic mechanisms are the primary drivers. The marginal increase from >98% at pH 6 to the maximum 99.41% at pH 8 represents the actual contribution of electrostatic attraction, which becomes operative above pH_pzc_ as a supplementary force.

#### 3.3.2. Effect of adsorbent dosage

The effect of PMC dosage on CV removal efficiency was investigated by varying the adsorbent dose from 10 to 100 mg under optimized experimental conditions, as shown in Fig. 7b. The effectiveness of dye removal was strongly correlated with the adsorbent dosage. PMC demonstrated significant dye uptake even at low adsorbent loading, with a removal efficiency of 96.08% at a low dosage of 10 mg. Due to the increased availability of active adsorption sites and improved dye–adsorbent interactions, increasing the adsorbent dosage to 20–40 mg yielded a marked increase in removal efficiency, reaching a maximum of 99.41% at 40 mg. Increasing the adsorbent dosage beyond 40 mg did not significantly enhance removal efficiency, with values remaining relatively constant at 99.13–99.30% across dosages from 50 to 100 mg. The aggregation of adsorbent particles and the existence of additional adsorption sites are responsible for this decline in behavior, resulting in site inefficiency at larger doses. Overall, the findings indicate that the optimal adsorbent dosage for achieving the highest CV removal efficiency is 40 mg of PMC.

#### 3.3.3. Effect of initial dye concentration

The effect of initial CV concentration on PMC adsorption efficiency was investigated over the range 20–100 mg L^−1^, as shown in Fig. 7c. PMC showed a removal efficiency of 99.76% at a dye concentration of 20 mg L^−1^. The removal efficiency, which varied from 99.18% to 99.45%, remained constant as the initial dye concentration increased from 40 to 80 mg L^−1^. This suggests that there are sufficient active adsorption sites on the PMC surface at lower dye loadings. A substantial decrease in removal efficiency was observed at 100 mg L^−1^, with the efficiency decreasing to 97.46%. The decrease in adsorption efficiency at higher initial dye concentrations is due to saturation of active sites on the PMC surface. Dye molecules readily interact with numerous surface functional groups at low concentrations, resulting in near-complete removal. At elevated concentrations, the number of dye molecules exceeds the adsorbent's adsorption capacity, resulting in incomplete uptake. The results indicate that PMC is effective for CV removal at low to moderate dye concentrations and remains effective at higher dye levels.

#### 3.3.4. Effect of contact time

The effect of contact time on the adsorption efficiency of CV onto PMC was investigated over 5–60 minutes under optimized experimental conditions, as shown in Fig. 7d. A significant improvement in dye removal efficiency was observed during the initial stages of adsorption, suggesting strong interactions between the dye molecules and the PMC surface. The removal efficiency was 72.76% after 5 minutes and increased gradually over the contact period, reaching 86.56% at 15 minutes and 91.40% at 20 minutes. The rapid initial adsorption is attributable to the large number of accessible active sites on the PMC surface. Due to the progressive occupation of active sites, the adsorption rate steadily reduced as the contact time increased. Equilibrium was attained within 50–60 minutes, yielding maximum removal efficiencies of 98.28% and 98.90%, respectively. The saturation of available adsorption sites and the attainment of adsorption–desorption equilibrium is indicated by the absence of discernible further improvement in dye removal beyond this point. The findings indicate that a contact time of 60 minutes is optimal for CV adsorption onto PMC.

#### 3.3.5. Effect of temperature

The effect of temperature on the adsorption performance of CV with PMC was studied over the temperature range 30–80 °C, as shown in Fig. 7e. The adsorption efficiency of PMC showed minimal dependence on temperature, indicating that thermal effects do not significantly affect dye–adsorbent interactions. PMC obtained a removal effectiveness of 98.66% at 30 °C, which slightly increased to a maximum of 99.11% at 40 °C. The greater accessibility of active adsorption sites at moderately elevated temperatures and the increased molecular mobility of dye molecules are responsible for this increase in efficiency. However, removal effectiveness gradually decreased with increasing temperature, reaching 96.79% at 50 °C and remaining above 98.20% at higher temperatures.

The weakening of physical interactions between CV molecules and the PMC surface, such as hydrogen bonding and van der Waals forces, may account for the small decrease in adsorption effectiveness at high temperatures. These findings suggested that the ideal temperature for CV adsorption with PMC was 40 °C.

### 3.4. Adsorption isotherms

**Fig. 8:**
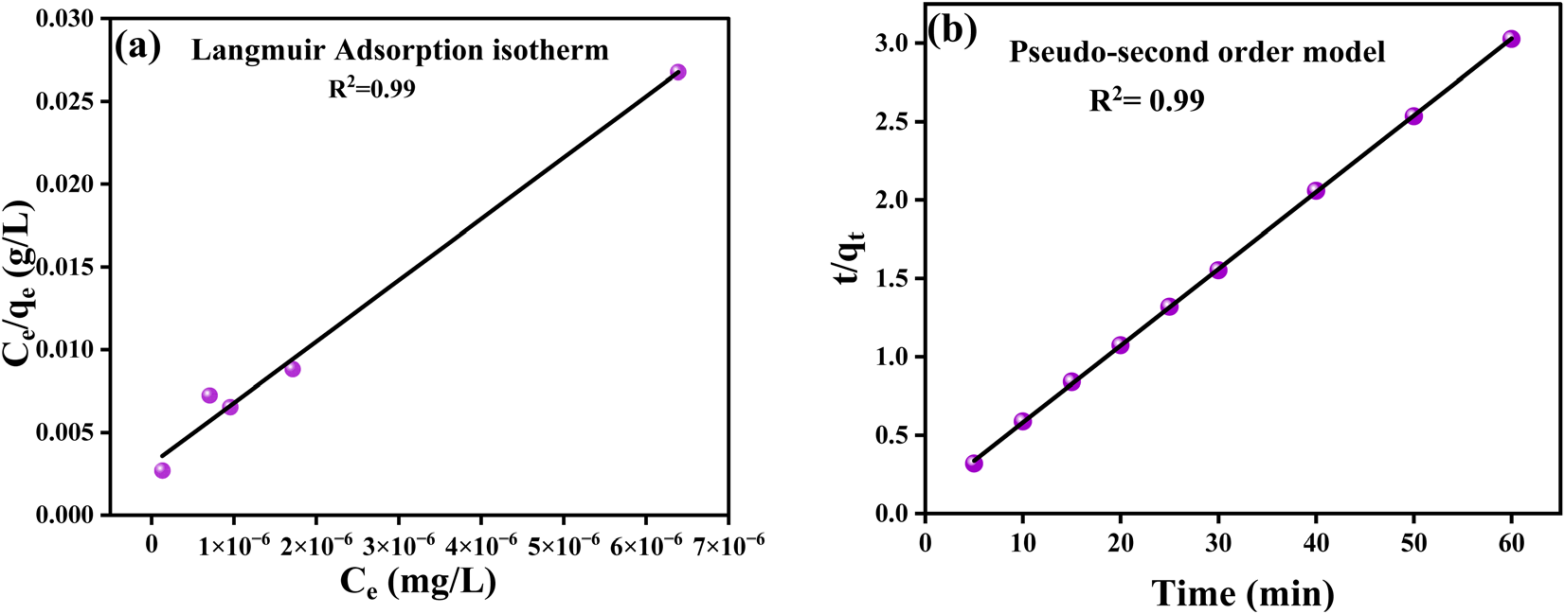
Adsorption isotherm models for CV (a) Langmuir adsorption isotherm model, and kinetic models (b) pseudo-second order model.

The Langmuir, Freundlich, Dubinin–Radushkevich (D–R), and Sips isotherm models were used to assess the equilibrium adsorption behavior of CV on PMC (Table 2). The Langmuir model is depicted in Fig. 8a, while the other models are given in the SI (Fig. S4–S6). The Langmuir equation had the highest correlation coefficient among the four models, indicating the best fit to the experimental data. This excellent fit indicates that monolayer coverage arises primarily from CV adsorption *via* homogeneous, nearly equivalent active sites on the PMC surface. The combined development of accessible micro–mesoporosity and abundant surface functional groups during KOH activation enhances the interaction between PMC and CV molecules, resulting in effective adsorption performance, despite the material's moderate specific surface area, as reflected by the Langmuir fitting parameters. In addition, the separation factor (*R*_L_) values for each beginning level were in the optimal range (0 < *R*_L_ < 1), indicating the spontaneity and efficiency of CV uptake. The adsorption equilibrium data were then evaluated using the Sips isotherm model to account for biomass-induced PMC surface energy heterogeneity. The Langmuir model showed a higher correlation coefficient (*R*^2^ = 0.99), indicating that CV adsorption on PMC followed monolayer behavior on homogeneous active sites, while the Sips model fit fairly well (*R*^2^ = 0.961). The Sips model revealed more about the PMC surface's adsorption capabilities (Fig. S6).

**Table 2:** Adsorption isotherms and kinetic models

Equilibrium model	Parameter	CV dye
Langmuir isotherm	*q*_m_ (mg g^−1^)	110.18
	*K*_L_ (L mg^−1^)	2.93
	*R* ^2^	0.99
Freundlich isotherm	*K*_F_ (mg g^−1^)	19.55
	*n*	2.327
	*R* ^2^	0.929
Dubinin–Radushkevich isotherm	*K*_d_ (kJ^2^ mol^−2^)	3.292
	*E* (kJ mol^−1^)	0.389
	*R* ^2^	0.944
Sips adsorption isotherm	*q*_m_ (mol g^−1^)	2.70 × 10^−4^
	*n*	1.05
	*K*_s_	6.34 × 10^5^
	*R* ^2^	0.961

**Kinetic models**		
Pseudo-second order model	*q*_e_(cal) (mg g^−1^)	20.4499
	*k*_2_ (g mg^−1^ min^−1^)	0.0257
	*R* ^2^	0.9998
Intraparticle diffusion model	*K*_id_ (min^0.5^)	0.7274
	*R* ^2^	0.875

On the other hand, the relatively lower *R*^2^ values found for the Freundlich and D–R models suggest that pore-filling mechanisms and surface heterogeneity have a lesser role in adsorption. All of these findings support the idea that the Langmuir model best describes the equilibrium interaction between CV and PMC.

### 3.5. Kinetic model

The adsorption kinetics of CV onto PMC were examined using the pseudo-second-order and intra-particle diffusion models, with fitting parameters detailed in Table 2 and corresponding plots shown in Fig. 8b and S7. The pseudo-second-order model provided a strong fit to the experimental data (*R*^2^ = 0.9998), and the calculated equilibrium adsorption capacity was in close agreement with the experimental value, highlighting the model's effectiveness in understanding the adsorption process. The dominant role of pseudo-second-order kinetics indicates that the rate-determining step is primarily governed by surface-controlled interactions, such as electrostatic attraction and non-electrostatic interactions between CV molecules and PMC. This finding is consistent with the rapid dye removal and strong adsorption capacity observed in tests. Although the intra-particle diffusion model demonstrated reasonable linearity (*R*^2^ = 0.875), deviations from ideal fitting and a nonzero intercept (Table 2) suggest that internal pore diffusion is not the sole governing mechanism. Rather, the adsorption process comprises several steps, including rapid adsorption on the external surface and slow diffusion into the adsorbent's porous structure. Based on the kinetic results, intra-particle diffusion is a secondary contributor to the surface interactions governing CV adsorption on PMC.

### 3.6. Preliminary adsorption test with coloured industrial wastewater

The evaluation of the practical applicability of the synthesized PMC, two coloured industrial dye wastewater samples (IW-1 and IW-2) were tested under realistic conditions. Before adsorption experiments, the samples were characterized for pH, total dissolved solids (TDS), and conductivity (Table S2). The industrial wastewater samples were used directly in the adsorption experiments after simple filtration to remove suspended particulate matter such as sludge, sand, and other solid impurities. No additional pre-treatment or pH adjustment was performed before the experiments. The adsorption process was monitored using UV-vis spectroscopy (Fig. S8a–d). For IW-1, a characteristic absorption band was observed at approximately 420 nm. Similarly, IW-2 exhibited a major absorption peak at approximately 727 nm. To verify that the observed colour removal was primarily due to adsorption, additional experiments were conducted under dark and sunlight conditions. Under dark conditions, IW-1 and IW-2 showed removal efficiencies of 96.9% and 87%, respectively, while under sunlight, the efficiencies were 98.5% and 90.6%. The small differences observed (1.6% for IW-1 and 3.6% for IW-2) indicate that photocatalytic degradation contributes minimally to the overall removal process, confirming adsorption as the dominant mechanism. Therefore, this study provides a preliminary qualitative and quantitative assessment of the material's decolorization efficacy in a complex wastewater matrix, rather than a conclusive investigation of specific dye species.

### 3.7. Adsorption mechanism

**Fig. 9:**
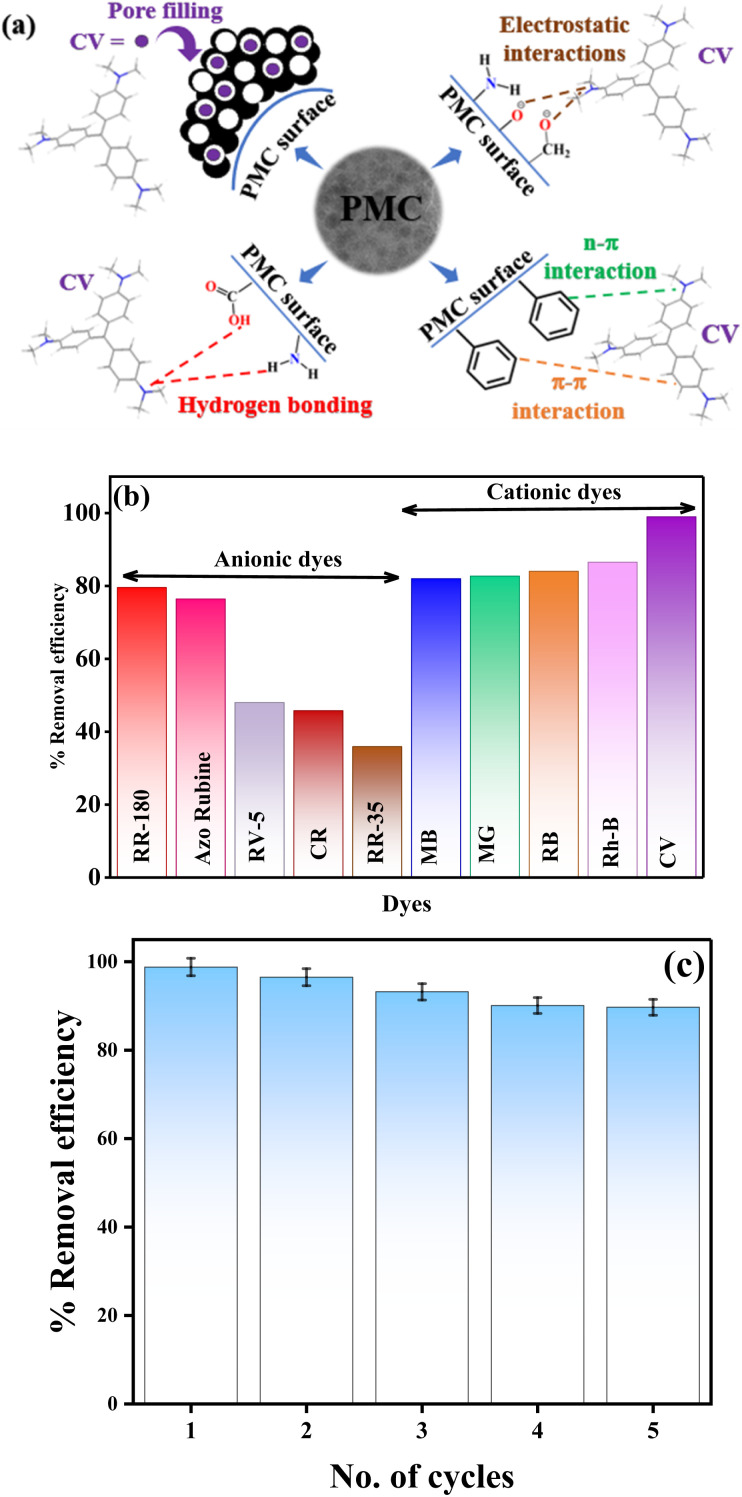
(a) Possible adsorption mechanism for the adsorption of CV by PMC adsorbent, (b) PMC adsorbent selectivity towards different cationic and anionic dyes (all dyes were tested at neutral pH), and (c) reusability of PMC adsorbent for the removal of CV.

The adsorption of CV onto PMC is governed by multiple synergistic interactions arising from the adsorbent's hierarchical porous structure and nitrogen-enriched carbon framework. Effective pore filling and access to internal adsorption sites are enabled by CV molecules that penetrate the interconnected porous network formed during KOH activation, which is responsible for the initial rapid uptake. This is followed by external mass transfer and intraparticle diffusion. The adsorption behaviour observed across a broad pH range indicates that both non-electrostatic and electrostatic interactions contribute to the adsorption of CV. At pH values above the pH_pzc_ (6.8), deprotonation of surface oxygen-containing groups generates a more negatively charged surface, as reflected by the zeta potential value of −35.6 mV at pH 7, thereby enhancing electrostatic attraction toward cationic CV molecules. However, the consistently high adsorption efficiencies observed near and below the pH_pzc_ indicate that electrostatic attraction is not the sole mechanism of adsorption. Nitrogen and oxygen-containing functional groups provide abundant active sites for adsorption through hydrogen bonding, while the conjugated carbon domains of PMC interact with the aromatic rings of CV through π–π stacking. In addition, the hierarchical porous structure facilitates pore filling and molecular diffusion. These non-electrostatic interactions contribute substantially to CV adsorption under neutral and weakly acidic conditions, whereas electrostatic attraction further enhances adsorption at alkaline pH. As shown schematically in Fig. 9a, CV adsorption on PMC occurs through a synergistic mechanism involving pore filling, electrostatic attraction, hydrogen bonding, and π–π interactions. Therefore, the high adsorption efficiency of PMC arises from the combined contribution of its porous structure, surface functionalities, and multiple complementary adsorption mechanisms.

### 3.8. Selectivity of dyes

The adsorption selectivity of PMC was systematically evaluated *via* a series of anionic (RR-180, Azo Rubine, RV-5, CR, and RR-35) and cationic (MB, MG, RB, Rh-B, and CV) dyes, as presented in Fig. 9b. PMC showed a significant affinity for cationic dyes, achieving substantially greater removal efficiencies than for anionic dyes. CV demonstrated the highest removal efficiency, approximately 98.9%, followed by Rh-B, RB, MG, and MB, each of which achieved 80% removal. The superior removal of CV is attributed to the favourable match between its molecular diameter (∼1.6 nm) and the average mesopore diameter of PMC (∼3.8 nm), facilitating efficient molecular diffusion and access to internal adsorption sites. Because the average pore diameter is more than twice the molecular diameter of CV, mass transfer within the porous network is expected to be governed predominantly by molecular diffusion rather than wall-controlled diffusion, thereby reducing pore-wall collisions and enhancing transport to internal adsorption sites.^30^ On the other hand, anionic dye adsorption was substantially decreased, especially for RR-35 and CR. The observed selectivity toward cationic dyes is enhanced by electrostatic attraction between the negatively charged PMC surface and positively charged dye molecules, whereas electrostatic repulsion disfavors the adsorption of anionic dyes. However, the higher adsorption of CV cannot be attributed to electrostatic interactions alone, as π–π stacking, pore accessibility, and hydrogen-bonding interactions also contribute to its adsorption affinity. In addition, the presence of oxygen-containing surface functional groups enhances interactions between the dye and the adsorbent *via* hydrogen bonding and π–π stacking, thereby facilitating the preferential adsorption of CV. These findings demonstrate PMC's excellent selectivity for CV and its potential to remove cationic dyes from contaminated water effectively.

### 3.9. Recyclability

Adsorbents' reusability is an essential factor for their practical use in wastewater treatment, as it directly affects operational costs and process sustainability. Through multiple adsorption–desorption cycles using CV dye under ideal conditions, the regeneration and reuse capability of the PMC adsorbent was tested. The CV-loaded PMC adsorbent was regenerated by sonication in ethanol for 30 minutes at room temperature after 60 minutes of adsorption tests. After regeneration, the adsorbent was reused for the next cycle. As the cycle number increased, PMC's adsorption efficiency gradually decreased, as seen in Fig. 9c. In the first cycle, the removal efficiency was 98.8%; after five cycles, it was 89.7%. Even after five consecutive cycles, PMC maintained high adsorption efficiency despite this minimal decrease, demonstrating excellent structural stability and regeneration capacity. These findings demonstrate the PMC adsorbent's potential for recurrent use in wastewater treatment systems and validate its successful reusability for CV removal.

### 3.10. Phytotoxicity study

**Fig. 10:**
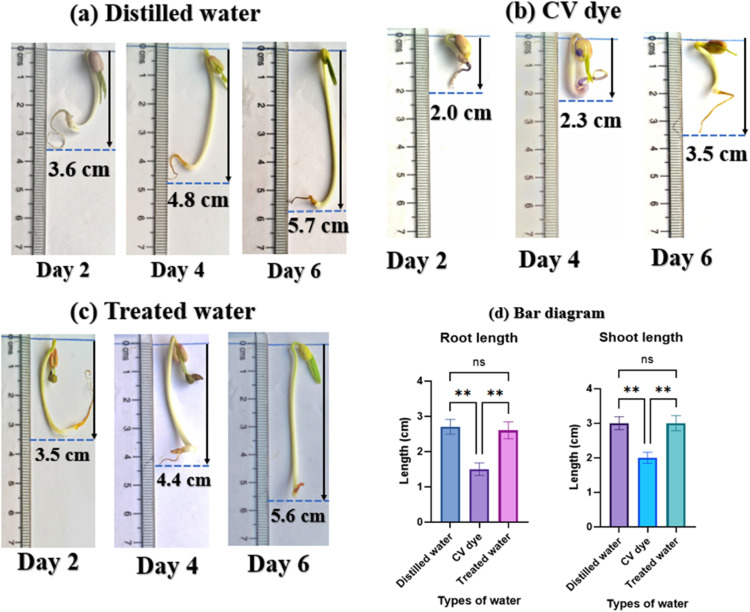
Phytotoxicity test of *Vigna radiata* seeds irrigated with (a) distilled water, (b) CV dye, and (c) treated water, (d) bar diagram showing seed germination growth in various water samples with respect to root and shoot lengths. Statistical significance was evaluated using one-way ANOVA. Data are expressed as mean ± SD (*n* = 3). **p < 0.05; ns = not significant.

To assess the ecological safety of CV-dye-contaminated water before and after treatment with the PMC adsorbent, a phytotoxicity study was conducted using *Vigna radiata* (mung bean), as shown in Fig. 10a–d. Key growth indicators included seed germination percentage, root length, and shoot length, with results compared with distilled water, which served as the negative control. The seeds that were watered with distilled water showed excellent growth and normal germination (100%) with average root and shoot lengths of 2.7 ± 0.21 cm and 3.0 ± 0.19 cm, respectively. On the other hand, exposure to untreated CV dye water significantly inhibited plant growth, resulting in a germination rate of 67% and a marked decrease in root length (1.5 ± 0.18 cm) and shoot length (2.0 ± 0.16 cm), indicating pronounced phytotoxic effects of the dye. Interestingly, seeds treated with CV water and PMC demonstrated significant recovery across all growth parameters. The germination rate increased to 83%, with root and shoot lengths of 2.6 ± 0.24 cm and 3.0 ± 0.22 cm, respectively, values close to those observed for the control group shown in Table 3. Visual observations conducted over 2, 4, and 6 days confirmed a progressive increase in growth in treated water, compared with the inhibited development observed in CV-dye water. These findings show that the phytotoxic effects of CV dye are successfully reduced by PMC treatment, which also restores plant growth and validates the treated effluent's beneficial environmental impact and removal potential.

**Table 3:** Phytotoxic study of *Vigna radiata* seeds

Parameter studied	Distilled water	CV dye	Treated water
Germination (%)	100	67	83
Length of root (cm)	2.7 ± 0.21	1.5 ± 0.18	2.6 ± 0.24
Length of shoot (cm)	3.0 ± 0.19	2.0 ± 0.16	3.0 ± 0.22

### 3.11. Comparison table

The comparative data in Table 4 clearly demonstrate that the activating agent strongly governs the adsorption capacity, equilibrium contact time, and specific surface area of adsorbents. Efficient adsorbate uptake is consistently associated with a well-developed porous network, a high specific surface area, and abundant surface functionalities capable of interacting with cationic dye molecules, regardless of the activation strategy employed. The use of acids, alkalis, salts, or heteroatom-rich precursors effectively tailors pore architecture and surface chemistry, thereby enhancing adsorption through electrostatic attraction, π–π interactions between aromatic domains, and pore-filling effects. Across all reported systems, activated or surface-functionalized materials exhibit higher adsorption capacities and shorter equilibrium times than their non-activated counterparts, underscoring the central role of activation in controlling adsorption kinetics and dye uptake. While several reported adsorbents exhibit superior maximum adsorption capacities, these performances are often associated with exceedingly high specific surface areas, prolonged equilibrium times, or assessments conducted under idealized single-component laboratory conditions that lack validation against real wastewater. PMC, by contrast, removes dyes quickly on a moderate surface area. The mesopore diameter of PMC (3.8 nm) closely matches the kinetic diameter of CV (1.6 nm), facilitating rapid molecular diffusion and explaining the 60 min equilibrium time despite lower surface area. By comparison, N-PPH (surface area = 3940 m^2^ g^−1^) and PPW with H_3_PO_4_ (surface area = 904.56 m^2^ g^−1^) require 50 and 180 min respectively, confirming that pore-molecule size compatibility governs adsorption kinetics more decisively than surface area alone.^31^ This indicates that optimized surface chemistry, a strong negative zeta potential, and super-hydrophilic properties can compensate for the lack of ultrahigh surface area by accelerating mass transfer and enabling selective electrostatic interactions. Overall, these results indicate that judicious selection and design of activating agents are critical for developing high-performance adsorbents with rapid kinetics, high uptake, and reliable reusability for the treatment of dye-laden wastewater.

**Table 4:** Comparative table of the other adsorbents *vs.* the prepared adsorbent^a^

S. no.	Adsorbent	Activating agent	Adsorbate	Surface area (m^2^ g^−1^)	Contact time	Adsorption capacity (mg g^−1^)	Ref.
1	PPW	H_3_PO_4_ + KOH (two-step activation)	Pb^2+^	833	60 min	9.3	32
2	PPW	K_2_CO_3_ activation	Sodium diclofenac	866	24 h	68.5	33
3	PP-AC	NaOH	Chromium(vi) ions	—	240 min	0.94	34
4	PPW	Activation with H_3_PO_4_	Cobalt ions	904.56	180 min	373	35
5	PP	—	Pb(ii), Cr(iii), Cd(ii) and Cu(ii)	—	60 min	14.93	36
						38.46	
						200.00	
						15.63	
6	PP	ZnCl_2_	Copper ions	1078	8 days	74	37
7	PP	—	Copper(ii)	—	20 min	0.3877	38
8	N-PPH	KOH, urea	Congo red (CR)	3940	50 min	400	39
9	PP-HYD-ox	KOH, HNO_3_	Pramipexole (Prami)	—	1440 min	66	40
10	PPP	—	CR	—	90 min	6.9	41
11	PPW	HCl	Acid blue 113	—	120 min	11.71	42
12	PP	Calcination/carbonization	Cibacron blue P3R	—	180 min	270.3	43
13	PP	—	CR	611.84	90 min	147.00	14
14	LIGPS50	ZnCl_2_	CR	126.69	15 min	278.65	44
15	PMC	Melamine and KOH	CV	284.303	60 min	110.18	This work

a Abbreviations: PPW – potato peel waste; PP-AC – potato peel activated carbon; PP – potato peel; N-PPH – nitrogen-rich potato peel hydrochar; PP-HYD-ox – potato peel hydrothermally treated and oxidized; PPP – potato peel powder; LIGPS50 – laser-induced graphene derived from potato skins; PMC-nitrogen-enriched, hierarchically porous activated carbon.

Table 5 confirms the superior performance of the PMC by benchmarking it against different adsorbents reported in the literature. PMC greatly outperforms similar materials such as mesoporous nitrogen-doped carbon and many reported biochars and activated carbons with a CV adsorption capacity of 110.18 mg g^−1^. This high uptake demonstrates how PMC's nitrogen-rich surface chemistry is incredibly effective at targeting cationic pollutants.

**Table 5:** Adsorption capacities for different types of adsorbents/pollutants reported in recent literature

S. no.	Adsorbent	Pollutant	Adsorption capacity (mg g^−1^)	Ref.
1	Magnetic-activated carbon	Brilliant blue dye	98.12	45
2	Eucalyptus graphitic activated carbon	Direct yellow 12 dye	42.01	46
3	Activated carbon	Methyl orange (MO)	113.63	47
4	Commercially available activated carbon	MO	100	48
5	*Z. mays* activated carbon	MO	66.2	49
6	Lemon peels-derived activated carbon	MO and methylene (MB)	33 and 38	50
7	Activated carbon based on mesquite sawdust	MB	1.76	51
8	Food waste-derived AC	MB	47.72	52
9	N-doped hydrochar	MB and congo red	57.52 and 62.19	53
10	N-doped carbons	Rhodamine B	100	54
11	Self-nitrogen-doped porous activated carbon	Acid brown 14	107.5	55
12	N-doped bagasse biochar	MB	84.2	56
13	Mesoporous palladium–nitrogen-doped carbon	CV	53.9	57
14	Algal-based magnetic biochar nanocomposite	Azocarmine G2	71.3	58
15	Bamboo biochar	Basic fuchsin	98.13	59
16	Phosphated biochar	Malachite green	32	60
17	K_2_CO_3_ modified sludge biochar	Reactive black 5	30.33	61
18	Biochar	MB and MO	104.5 and 98.7	62
19	Nitrogen-enriched, hierarchically porous activated carbon	CV	110.18	This work

## 4. Conclusion

This study demonstrates the conversion of industrial potato peel waste into a nitrogen-doped activated carbon *via* melamine impregnation and KOH activation. The resulting material has a hierarchical porous structure (surface area ∼ 284 m^2^ g^−1^) and abundant N/O functional groups, resulting in a strongly negative surface charge (zeta ≈ −35.6 mV; pH_pzc_ ≈ 6.8). These features enable rapid and selective adsorption of the cationic dye CV through enhanced surface accessibility and electrostatic control, achieving equilibrium in ∼60 minutes with ∼98.9% removal (Langmuir *q*_m_ ≈ 110.18 mg g^−1^). The adsorption follows pseudo-second-order kinetics, indicating that surface interactions (electrostatic attraction, hydrogen bonding, π–π stacking) govern the uptake. PMC also effectively decolorized real textile effluents (>88% removal within 1 h) and retained ∼90% of its capacity after five ethanol regeneration cycles. Phytotoxicity tests show that treated water supports plant growth almost as well as pure water. These results demonstrate that N-doped potato peel carbon is a low-cost, sustainable adsorbent for the selective removal of cationic dyes from wastewater. More broadly, this work establishes a blueprint for upgrading food-processing residues—often overlooked due to perceived homogeneity—into high-performance separation materials. By coupling supply-chain realism with synthetic precision, PMC exemplifies a circular-economy adsorbent tailored for practical deployment. While this study establishes fundamental adsorption parameters under batch conditions, continuous-flow column studies represent an important direction for future work prior to field-scale deployment.

## Author contributions

M. Bhavani Lakshmi carried out all experimental work and prepared the original manuscript draft. Paramita Pattanayak and Tanmay Chatterjee performed data acquisition. Alibasha Akbar contributed to the writing, review, and editing of the manuscript. Mihir Ghosh conceived the research concept and provided overall supervision and guidance for the study.

## Conflicts of interest

There are no conflicts to declare.

## Supplementary Material

RA-OLF-D6RA02967A-s001

## Data Availability

All data supporting the conclusions of this study are available from the corresponding author upon reasonable request. The authors confirm that all relevant data are included within the article and its supplementary information (SI), with additional materials available upon request. Supplementary information is available. See DOI: https://doi.org/10.1039/d6ra02967a.
