# Supplementary material for: Upcycling factory potato peel into a hierarchically porous carbon for dye removal, phytotoxicity mitigation, and wastewater remediation
Source: RSC Adv. 2026 Jul 13;16(37):40040–57. doi: 10.1039/d6ra02967a (PMC13359596; doi:10.1039/d6ra02967a)
Supplement: Supplementary file 1 [file RA-016-D6RA02967A-s001.pdf]

Supplementary Information (SI) for RSC Advances.
This journal is © The Royal Society of Chemistry 2026

Supplementary information

Upcycling factory potato peel into a hierarchically porous carbon for dye removal, phytotoxicity mitigation, and wastewater remediation

M. Bhavani Lakshmi [a], Alibasha Akbar [a], Paramita Pattanayak[b], Tanmay Chatterjee [b], Mihir Ghosh [a*]

[a] Department of Chemistry, SRM Institute of Science and Technology, Kattankulathur 603203, Tamil Nadu, India

[b]Department of Chemistry, Birla Institute of Technology and Science, Pilani – Hyderabad Campus, Jawahar Nagar, Kapra Mandal, Hyderabad, Telangana 500078, India

**Corresponding author E-mail address: mihirg@srmist.edu.in*

1. Methods:

1.1 Acid/base titrations (Boehm's titration)

The surface characteristics (acidic or basic) of the nanohybrid materials were evaluated using Boehm's selective acid/base neutralization method.

The number of acidic groups on the activated carbon surface was determined using the Boehm titration method. A 0.1 M NaOH solution (50 mL) was placed in a conical container with 0.1 g of activated carbon sample. The conical bottle was subjected to oscillation in a shaker for 24 h at 25 °C (160 rpm) concurrently with a blank experiment at the same conditions. After oscillation, the solution was filtered, and 10 mL of the filtrate was titrated using HCl (0.1 M) and phenolphthalein as the indicator. Furthermore, the NaOH solution was replaced with $NaHCO_3$ solution (0.1 M) and Na_2CO_3 solution (0.1 M) to repeat the oscillation and filtering procedures. Hydrochloric acid (0.1 M) was used during titration, with bromocresol green-methyl red serving as the indicator. NaOH neutralizes the carboxyl, lactone, and phenolic hydroxyl groups, $NaHCO_3$ neutralizes the carboxyl group; and Na_2CO_3 neutralizes the carboxyl group and lactone group. The number of oxygen-containing functional groups was computed based on the amount of alkali solution consumed during the reaction. The functional groups(mmol/g) were determined using the following formula:

$$Functional\ groups\ (mmol/g) = \frac{(Normality \times Volume_{consumed})}{Molecular\ weight} * \frac{V_{initial}}{V_{titrated}}$$

N represents the normality of the titrant; $V_{consumed}$ denotes the volume of the titrant consumed; $V_{initial}$ denotes the total volume of the solution; $V_{titrated}$ is the volume taken for titration; and M.W denotes the molecular weight of the titrant.

1.2 Determination of pHpzc:

The pHpzc was determined to examine the surface charge of the activated carbon. The solid-addition technique was used to determine the point of zero charge (pHpzc) of the adsorbent. A 45 mL solution of 0.1 mol L^{-1} NaCl was transferred to a set of 100 mL conical flasks. The initial pH values of the solution were adjusted from 1.0 to 11.0 by the addition of either 0.1 mol L^{-1} HCl or 0.1 mol L^{-1} NaOH solutions. The solution volume in each flask was precisely adjusted to 50 mL by adding NaCl solution. Subsequently, 0.1 g was added to each flask, and the mixtures were stirred at 150 rpm. The final pH values (pH_f) of the solutions were determined after 48 h. The difference between the initial and final pH values ($\Delta pH = pH_i - pH_f$) was plotted against pH. The pHpzc was determined at the point where the resultant curve intersected the abscissa, when $\Delta pH = 0$.

1.3 Zeta potential vs pH

The zeta potential of PMC in pure water was assessed using a zeta potential analyzer (Zetasizer, Malvern Instruments, Worcestershire, UK, model Nano ZS) at different pH levels. The activated carbon suspension was diluted with water and sonicated for 30 min in an ultrasonic bath. The USC-1400 model operated at a distinct frequency of 40 kHz within the ultrasound spectrum. A Malvern 3000 Zetasizer NanoZS (Malvern Instruments, Malvern, UK) was used to measure the average particle size of the activated carbon. Particle diffusion under Brownian motion was measured, and the data were subsequently translated into particle size and size distribution using dynamic light scattering. It is employed in laser Doppler microelectrophoresis to create an electric field for the dispersion of the activated carbon suspension.

1.4 Freundlich adsorption isotherm:

The Freundlich model, describing multilayer adsorption on heterogeneous surfaces, is represented by:

$$\ln q_e = \ln K_f + \frac{1}{n}\ln C_e \tag{1}$$

where K_f and n Freundlich constants are indicative of adsorption capacity and intensity. From the plot of $\log q_e$ versus $\log C_e$, the values of $n = 2.327$ and K_f (Table 2) confirm favorable and intensive adsorption $(n > 1)$ as shown in Figure S4.

1.5 D-R adsorption isotherm:

The Dubinin–Radushkevich (D–R) isotherm (Figure S5), based on a Gaussian energy distribution, provides insight into the adsorption nature by estimating the mean free energy E

$$E = \frac{1}{\sqrt{2K_d}} \tag{2}$$

where K_d ($mol^2\ kJ^{-2}$) is the D–R constant. The parameters and R^2 values are summarized in Table 2.

1.6 Sips adsorption isotherm

The Sips isotherm model, which combines the characteristics of both Langmuir and Freundlich models and describes adsorption on heterogeneous surfaces (Figure S6), is represented by:

$$\ln\left(\frac{q_e}{q_m - q_e}\right) = \frac{1}{n} ln\ C_e + \ln K_s$$

where K_s, q_m, and n are the Sips constants related to adsorption affinity, maximum adsorption capacity, and surface heterogeneity, respectively as shown in Table 2. From the plot of $\ln\left(\frac{q_e}{q_m - q_e}\right)$ versus $\ln C_e$, the obtained R^2 value of 0.961 indicates reasonably good agreement with the experimental data.

1.7 Intraparticle diffusion model:

The intraparticle diffusion model, as proposed by Weber and Morris, evaluates the diffusion rate of adsorbate molecules within the pores of the adsorbent. It is expressed by:

$$q_t = k_i \times\ t^{0.5} + I \tag{4}$$

where q_t is the adsorption capacity at time t, k_i is the intraparticle diffusion rate constant (mg g^{-1} $min^{-0.5}$), and C represents boundary layer thickness. The rate constant k_i is obtained from the slope of q_t versus $t^{0.5}$ plots (Figure S7), with corresponding values summarized in Table 3. A correlation coefficient $R^2 = 0.875$ indicates that while intraparticle diffusion also significantly contributes to the adsorption kinetics, it is not the major rate-limiting step. But the pseudo-second order kinetics plays a major role in the adsorption, as well as intraparticle diffusion, which supports the kinetics in the adsorption of Crystal violet. The intercept C implies additional resistance from the external liquid film, revealing that both pore diffusion and boundary layer effects concurrently influence the overall adsorption rate.

1.8 Phytotoxicity test

The early seed's development and the development of the first structures are essential processes that regulate seed germination and the initial growth of seedlings. Germination assays were performed using *Vigna radiata* (mung bean) seeds to assess the phytotoxicity of CV and the treated water. The toxicity of untreated CV solution, PMC-treated CV solution, and the control sample (distilled water) was evaluated to investigate their effects on seed germination and seedling development. The phytotoxicity experiment was conducted according to a standard

method [55]. Sterilized Vigna radiata seeds were placed in Petri dishes lined with filter paper and moistened by using distilled water (control), a CV dye solution (20 mg L^{-1}), or an PMC-treated CV solution. The Petri dishes were incubated under controlled conditions for 6 days, after which the germination percentage, root length, and shoot length were measured to assess phytotoxic effects.

$$\text{Germination rate (\%)} = \frac{No.0f\ seeds\ germinated * 100}{Total\ number\ of\ seeds\ germinated}$$

Figures:

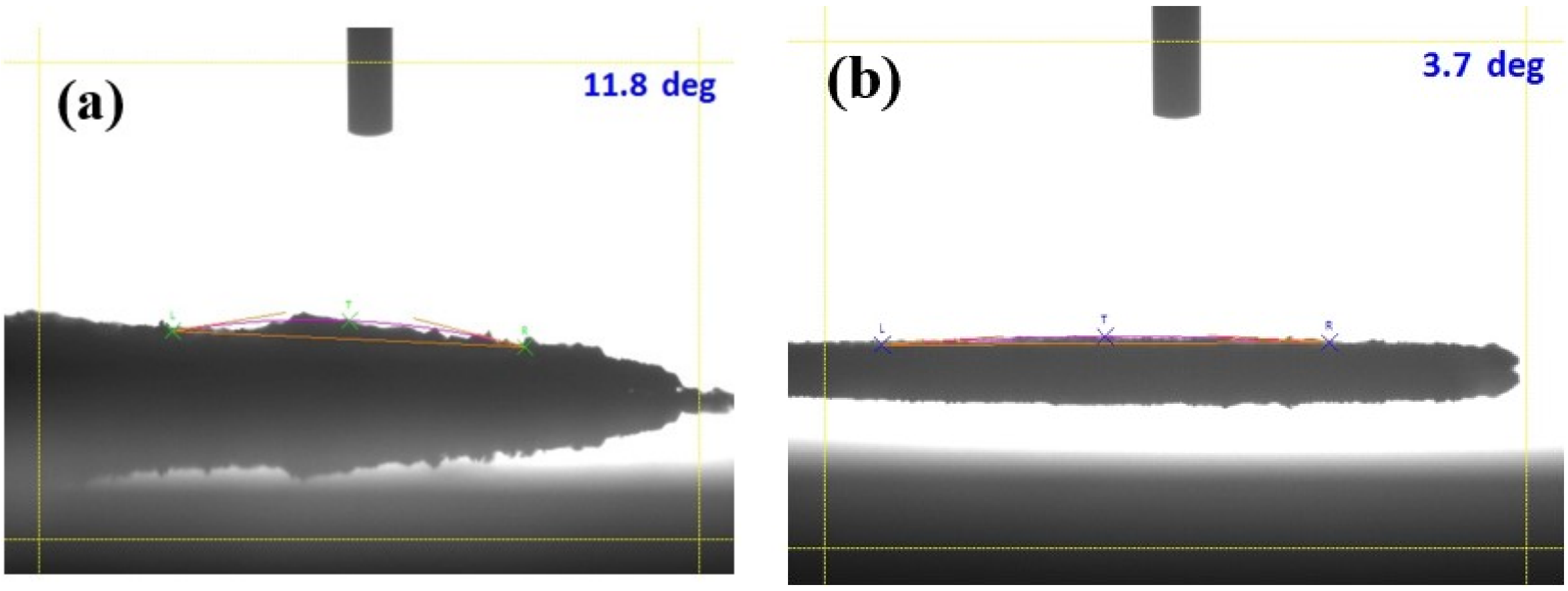

Figure S1: Contact angle measurement of (a) ST (hydrophilic), and (b) PMC (superhydrophilic).

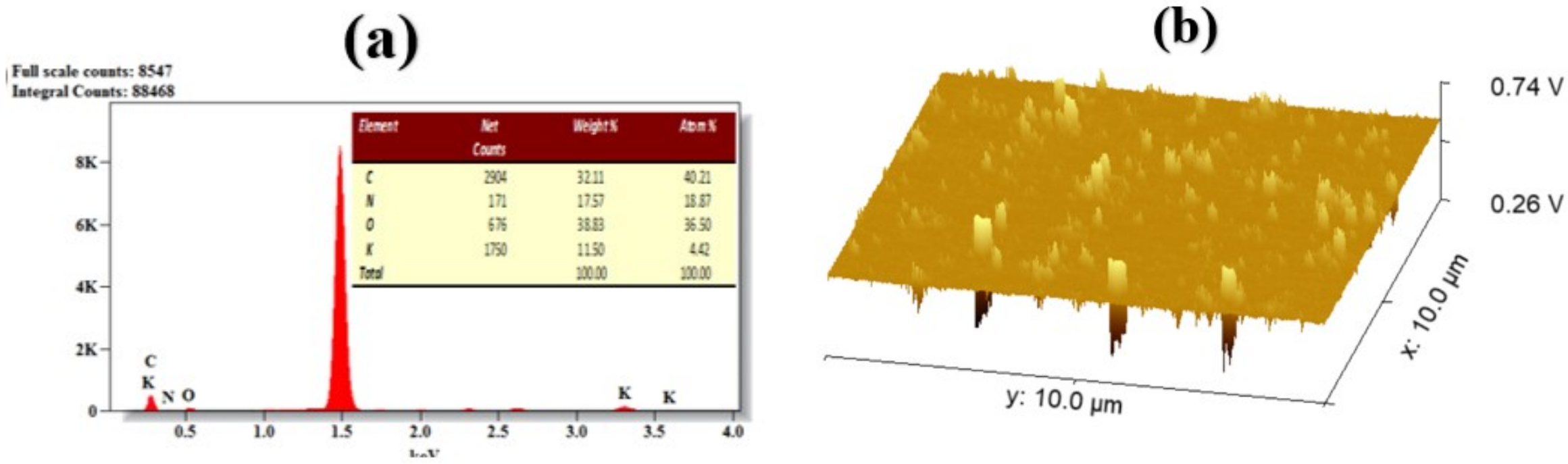

Element	Net Counts	Weight %	Atom %
C	2904	32.11	40.21
N	171	17.57	18.87
O	676	38.83	36.50
K	1750	11.50	4.42
Total		100.00	100.00

Figure S2: (a) EDS spectra of the PMC sample taken from the SEM. (b) AFM image of PMC sample.

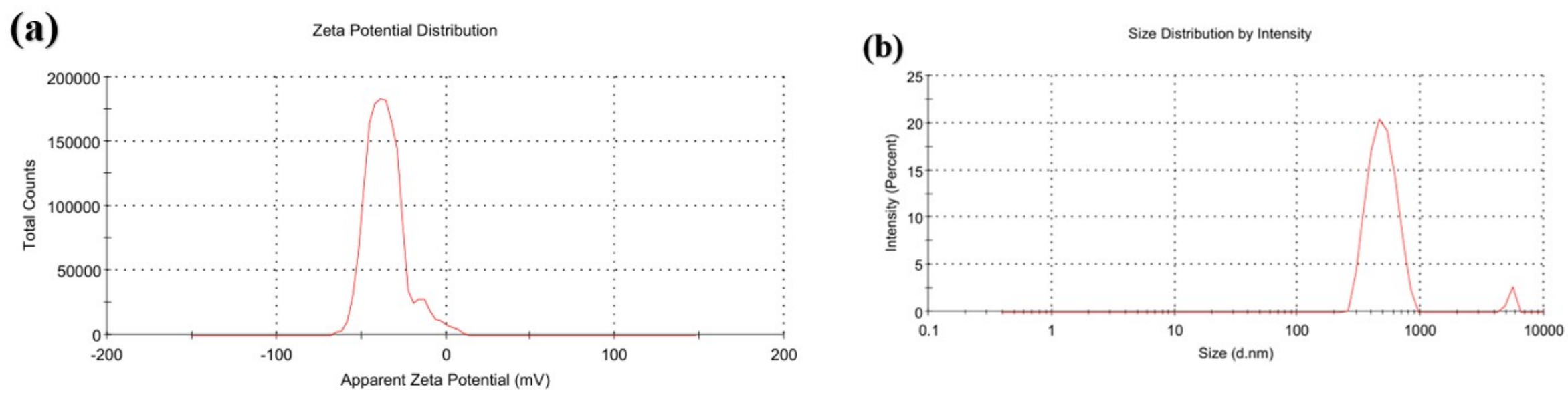

Figure S3: (a) Zeta potential of PMC and (b) Particle size distribution of PMC.

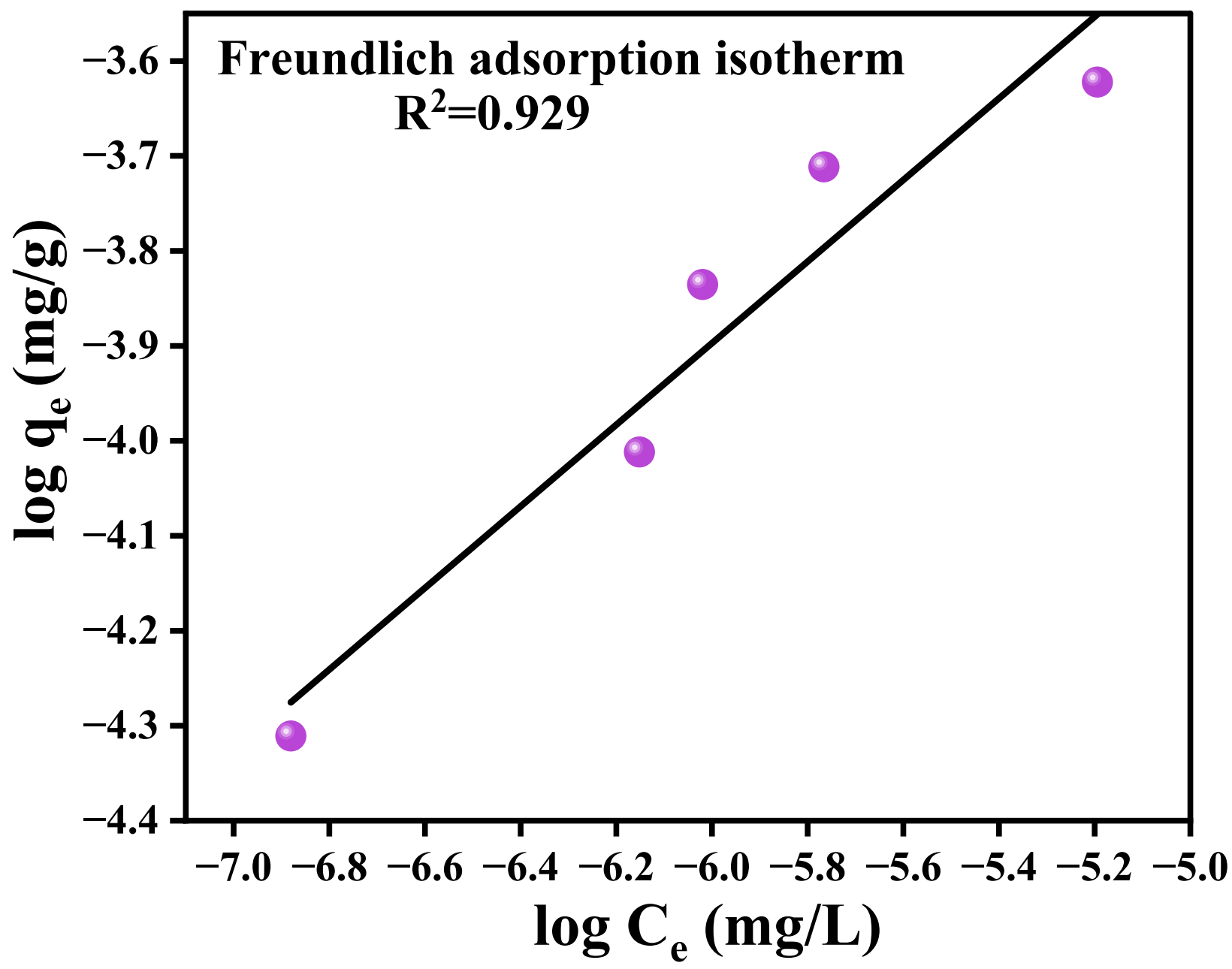

Figure S4: Freundlich adsorption isotherm model for PMC sample

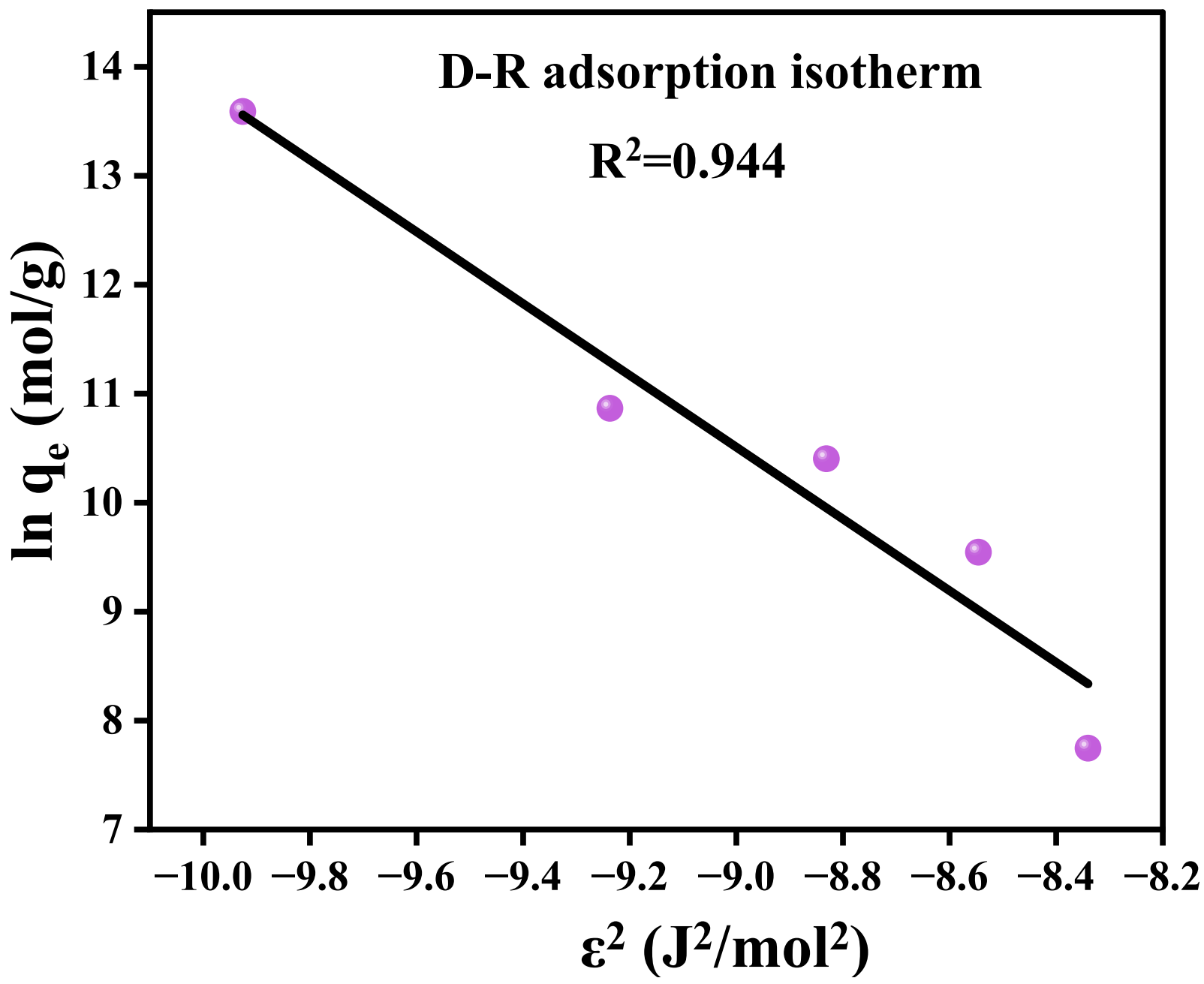

Figure S5: D-R adsorption isotherm model of PMC sample

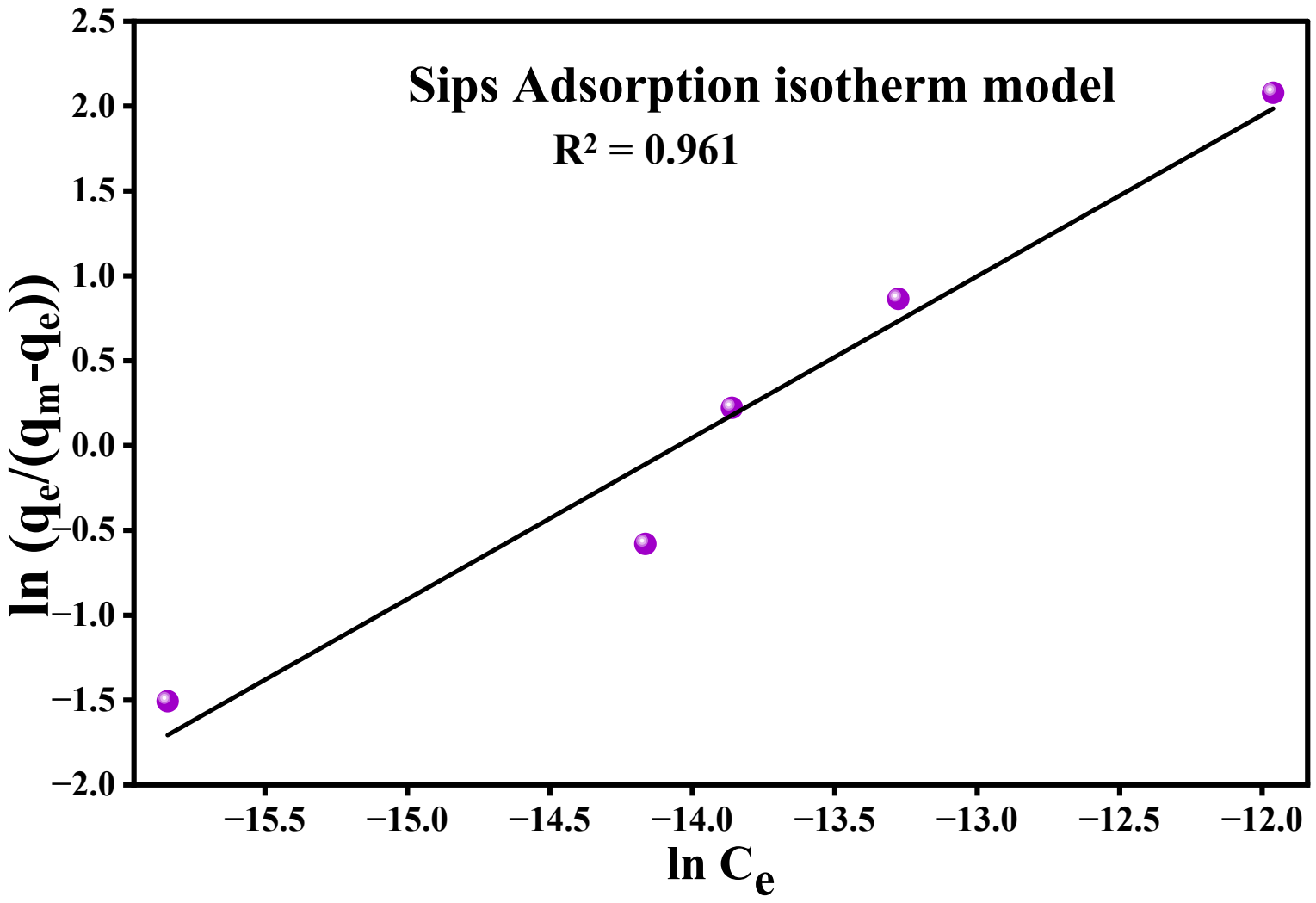

Figure S6: Sips Adsorption isotherm model

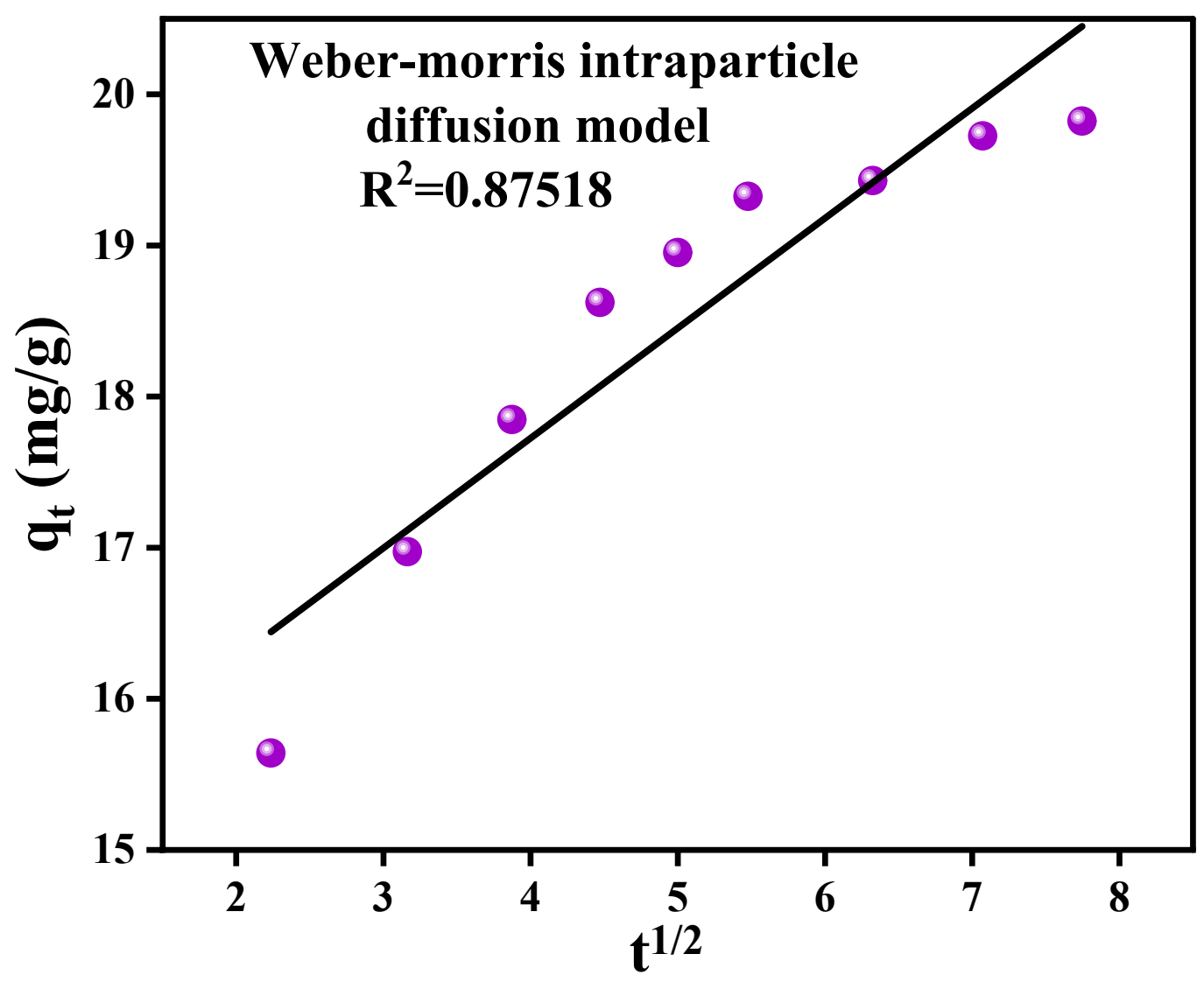

Figure S7: Weber-Morris intraparticle diffusion model for the PMC sample

Table S1: Proximate analysis of Potato peel waste.

**Component**	**Composition / Value**
Moisture content (%)	8.5
Ash content (%)	9.79
Volatile matter (%)	72.4
Fixed carbon (%)	9.3
Carbon (C, %)	43.78 ± 0.15
Hydrogen (H, %)	5.96 ± 0.12
Nitrogen (N, %)	4.06 ± 0.01
Oxygen (O, %)	46.21 ± 0.28
Starch (%)	16.83 ± 0.52
Nonstarch glucan (%)	7.75 ± 0.68
Galactan (%)	6.91 ± 0.10
Mannan (%)	0.75 ± 0.07
Xylan (%)	3.52 ± 0.21
Arabinan (%)	3.53 ± 0.06

Nonstarch polysaccharide (%)	22.46 ± 0.79
Acid-insoluble lignin and suberin (%)	15.94 ± 0.04
Acid-soluble lignin (%)	5.70 ± 0.20
CH_2Cl_2 extractives (%)	1.98 ± 0.15
Calorific value (MJ kg^{-1})	17.37 ± 0.38

Table S2: Industrial Wastewater (IW-1 and IW-2) Physicochemical characterization.

Sample	pH	TDS (ppm)	Conductivity (μS/cm)
IW-1	8.17	2197	4114
IW-2	8.08	964	2690

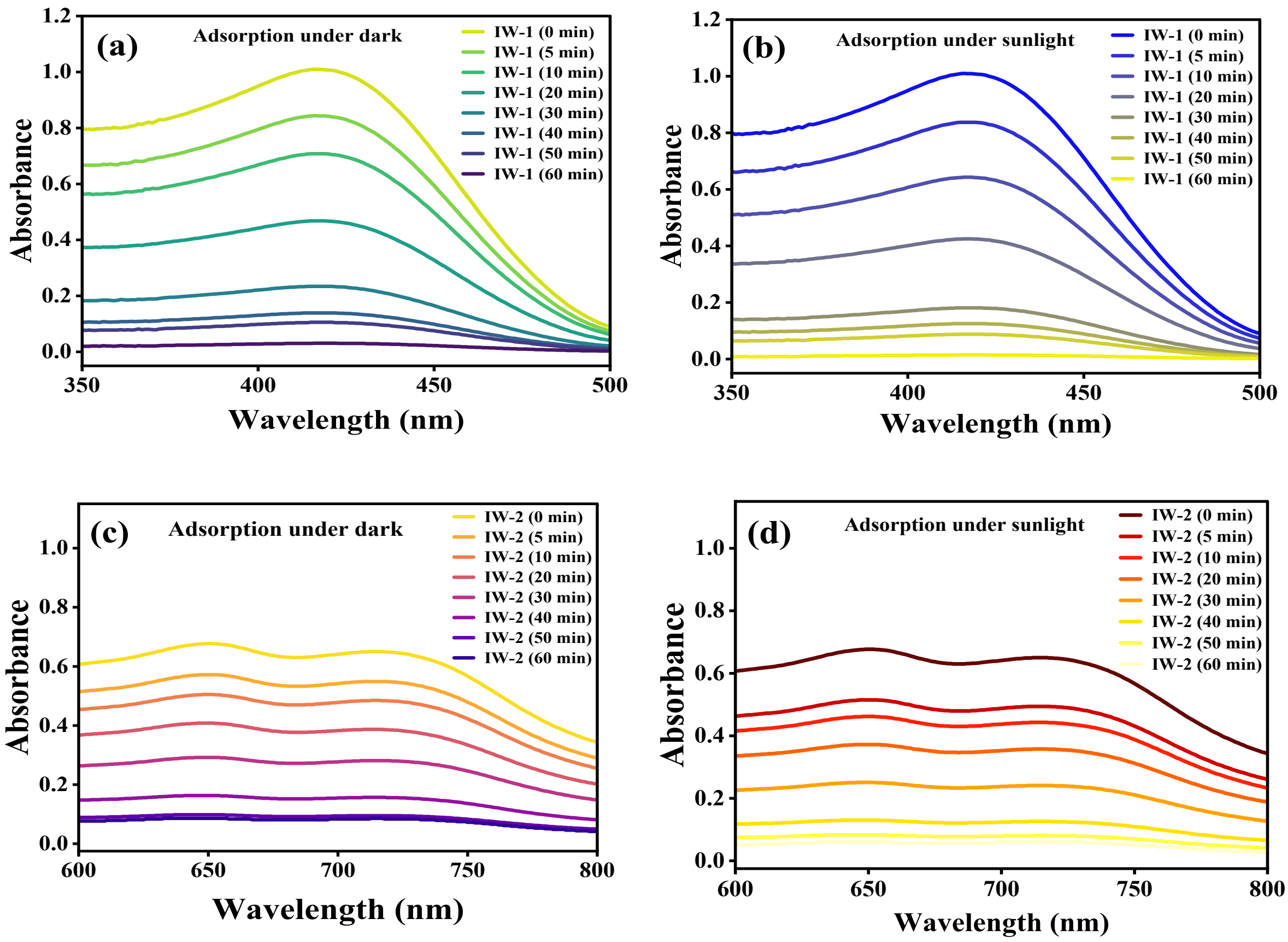

Figure S8. Adsorption experiments conducted under dark and sunlight conditions: (a) and (b) for IW-1, (c) and (d) for IW-2.
